# Tuning Defects in Ni‐Doped Maghemite for Enhanced Solar Driven Oxygen Evolution: Insights From *Operando* X‐Ray Spectroscopies

**DOI:** 10.1002/advs.202524386

**Published:** 2026-03-31

**Authors:** Francesco Paparoni, Hélène Magnan, Antoine Barbier, Andrea Zitolo, AndreaDi Cicco, Javad Rezvani, Emiliano Fonda

**Affiliations:** ^1^ Synchrotron SOLEIL L'Orme des Merisiers Gif‐sur‐Yvette France; ^2^ Sez. Fisica, Scuola di Scienze e Tecnologie Universitá di Camerino Camerino Italy; ^3^ Service de Physique de l'Etat Condensé CEA, CNRS Université Paris‐Saclay Gif‐sur‐Yvette France

**Keywords:** defects engineering, Fe_2_O_3_, OER photocatalysis, operando XAS

## Abstract

Maghemite (γ‐${\mathrm{Fe}}_{2}$ $O_{3}$) is a promising non‐precious‐metal‐containing photocatalyst for water oxidation (OER). Despite being less studied than Hematite, it offers similar corrosion resistance and a favorable band structure, along with higher conductivity and the advantage of an adaptable spinel structure. Its catalytic performance can be optimized by tuning the concentration of a donor dopant. In this work, we synthesized via molecular beam epitaxy ultra‐thin (6 nm) Ni‐doped γ‐${\mathrm{Fe}}_{2}$ $O_{3}$ (111) films with different Ni concentrations. We present a comprehensive study that sheds light on the structural reconfigurations induced by tuning the Ni concentration and the resulting effects on the OER onset and photoconversion efficiency in alkaline media. Samples' electronic reconfiguration under *operando* conditions and the role of Ni dopant on the catalytic mechanism are studied with X‐ray Absorption Spectroscopy. Light‐driven surface chemistry modifications during OER are probed with a novel X‐ray fluorescence *pump‐and‐probe* approach: Fixed energy X‐ray absorption PhotoVoltammetry.

## Graphical abstract

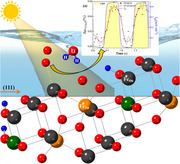

## 1 Introduction

Hydrogen is one of the most promising green energy carriers, since it has the highest specific energy of any currently employed fuel [1, 2]. It can be produced without greenhouse gas emissions through electrolysis to store the excess energy generated by intermittent renewable sources. However, according to the latest review published by the International Energy Agency (IEA), less than 1% of the 100 Mt of $H_{2}$ produced in 2024 was generated by low‐emission processes [3]. This is mainly due to the significant electrical input required to perform electrolysis and the high cost of the electrolyzer. Indeed, the hydrogen evolution reaction (HER) has a necessary oxidative counterpart, where molecular oxygen is evolved from water (OER) through a complex four‐electron process with the highest overpotential. Best catalysts for OER contain rare materials (e.g., Ru, Ir) and work best in aggressive alkaline electrolytes [4]. Nonetheless, electrochemical technologies are growing rapidly, as demonstrated by the 30% increase in low‐emission $H_{2}$ global production between 2024 and 2025 [3]. Among the green sources, solar energy stands out as the most abundant renewable energy source on Earth, with an average annual solar power of ∼ 36000 TW·yr that reaches our planet's surface [5]. This energy can be exploited to fuel electrolysis through photovoltaic panels. However, a direct solar‐to‐chemical energy conversion is an attractive possibility, allowing device simplification and limiting intermediate losses [6]. This can be accomplished in photoelectrochemical (PEC) cells; when a photon is absorbed by a semiconductor, it generates an electron/hole pair. If the photon energy is more energetic than the semiconductor bandgap, it can promote the electron to the conduction band, leaving a hole in the valence band (VB). Such photogenerated charges are metastable and typically require an electric field to sustain charge separation. The charge formation can stably occur within a superficial region of the photoelectrode known as the space charge layer, whose thickness depends on the material properties and applied bias. In an n‐type semiconductor, electrons are drawn to the bulk while holes accumulate toward the electrode surface upon illumination. If the band alignment conditions with the electrolyte are respected, the accumulated charges can be injected into the electrolyte, catalyzing the water splitting reaction. At the current state of the art, PEC cells are not industrially competitive to photovoltaic tandem systems, since real PEC devices struggle to surpass 5% STH (Solar‐to‐Hydrogen) conversion efficiency, remaining well below the 10% industrially required target [7]. Despite their currently low efficiency, PEC cells represent a young technology with substantial room for development. Most efforts currently focus on OER catalysts, which are regarded as bottlenecks in electrochemical technologies. Several transition metal oxide systems have shown promising performances, proving how, upon precise tuning of the material electronic structure, surface terminations, and binding strength with the reaction intermediates [8] steps toward cost‐effective photoanodes are possible.

Trivalent iron oxide systems like hematite (α‐${\mathrm{Fe}}_{2}$ $O_{3}$) are among the most studied materials for solar‐driven OER in alkaline media, due to optimal VB position, bandgap size, photochemical stability, and earth abundance [9, 10], which theoretically allows to achieve the highest solar‐to‐hydrogen conversion efficiency (15%) [11, 12, 13]. Despite such high promises, real hematite electrodes show low energy conversion efficiency, as a consequence of a very short hole diffusion length (2 ‐ 4 nm), lifetime (3–10 ps) combined with scarce surface catalytic activity. It has been shown that hematite conductivity and carrier's diffusion length can be increased by n‐type doping with a secondary transition metal [14, 15, 16]. Precise doping engineering studies are required to maximize the beneficial effects of the dopant element, since an excessive dopant concentration can lead to trap states formation and facilitate charge recombination. Maghemite (γ‐${\mathrm{Fe}}_{2}$ $O_{3}$) is an attractive alternative. Is the second most stable trivalent iron oxide polymorph and it exhibits a similar bandgap (2 ‐ 2.2 eV [17]) while having an inverse spinel structure ${\mathrm{AB}}_{2}$ $O_{4}$, with A and B being ${\mathrm{Fe}}^{3+}$ cations in tetrahedral (${\mathrm{Fe}}_{Td}$) and octahedral (${\mathrm{Fe}}_{Oh}$) sites, respectively. γ‐${\mathrm{Fe}}_{2}$ $O_{3}$ has a similar structure compared to magnetite (${\mathrm{Fe}}_{3}$ $O_{4}$), but given the absence of ${\mathrm{Fe}}^{2+}$ sites, the charge neutrality is granted by the presence of 1/6 of ${\mathrm{Fe}}_{Oh}$ vacancies. Hence, the unit cell can be written as ${\left[{\mathrm{Fe}}^{3+}\right]}_{8,Td}{\left(\frac{5}{6}\left[{\mathrm{Fe}}^{3+}\right]\frac{1}{6}\left[□\right]\right)}_{16,Oh}O_{32}^{2-}$, with □ being an iron vacancy [18]. Compared to hematite, this structure offers higher conductivity and more facile and stable adjustment of composition by doping, thanks to the versatile spinel structure. Nonetheless, maghemite photoanodes have been investigated far less extensively than hematite, due to the metastability of γ‐${\mathrm{Fe}}_{2}$ $O_{3}$, which tends to convert to α‐${\mathrm{Fe}}_{2}$ $O_{3}$ at room temperature [19, 20, 21], complicating the synthesis process as well as a precise characterization of the catalyst. For this reason, maghemite PEC‐OER performances are still debated, with recent studies even suggesting they are completely hampered by a high defect concentration that facilitates charge‐recombination [22]. However, the limited control over sample crystallinity, phase purity and surface termination inherent to similar hydrothermal synthesis processes challenges the generalization of these findings. The phase stability of maghemite thin films on a metallic substrate and the resulting surface termination were theoretically investigated in recent works [23, 24]. These results demonstrated remarkable phase stability and adhesion of a (111) γ‐${\mathrm{Fe}}_{2}$ $O_{3}$ film on Pt (001).

Doping and surface termination control can improve maghemite PEC performance. For instance, density functional theory (DFT) results show that distinct γ‐${\mathrm{Fe}}_{2}$ $O_{3}$ surface terminations offer different active site densities and adsorbate attachment strength, with the (111) surface showing the best attachment energy and accessible iron sites [25]. At the same time, Nickel is a promising dopant element for maghemite: ${\mathrm{Ni}}^{2+}$ doping increases ${\mathrm{Fe}}_{2}$ $O_{3}$ conductivity and carrier concentration [26, 27]. The concurrent formation of donor states within the bandgap narrows the distance between the Fermi level and the conduction band, possibly improving visible light absorption and optimizing solar‐to‐hydrogen conversion efficiency. Moreover, minimal distortion of the cubic structure can be expected in the case of ${\mathrm{Fe}}_{Oh}$ replacement, since octahedrally coordinated ${\mathrm{Ni}}^{2+}$ and ${\mathrm{Fe}}^{3+}$ possess very similar ionic radii (0.69 and 0.65 Å respectively). The possibility of synthesizing a pure or precisely doped maghemite film with a well defined electrolyte/oxide interface, combined with the superior chemical stability and conductivity of a Pt substrate in alkaline media, offers an exceptional case study to investigate the PEC performances of this polymorph and optimize these parameters toward a more efficient and stable photoanode than better‐known hematite counterparts.

In this work, ultra‐thin Ni‐doped γ‐${\mathrm{Fe}}_{2}$ $O_{3}$ films are grown on Pt (001) single crystals, achieving epitaxial growth with preferential orientation along the (111) crystallographic plane. Samples with different Ni concentrations are compared. Crystallographic orientation and morphology were studied with Reflection High Energy Electron Diffraction (RHEED) and Atomic Force Microscopy (AFM). The effect of the Ni doping on the sample's structure is discussed via X‐ray Photoelectron Spectroscopy (XPS) and X‐ray Absorption Spectroscopy (XAS) in section 3, while the resulting PEC performances are discussed in Section 4 via Linear Sweep Voltammetry (LSV), Chronoamperometry (CA) and Incident Photon to Current Conversion Efficiency (IPCE). Structural reconfiguration under *operando* conditions is discussed using XAS and a novel pump‐and‐probe X‐ray fluorescence approach in Section 4.1.

## 2 Experimental Method

6 nm thick Ni‐doped γ‐${\mathrm{Fe}}_{2}$ $O_{3}$ (111) films were epitaxially grown on Pt (001) single crystal substrates (⌀ = 1 cm, thickness 1 mm) via oxygen plasma‐assisted molecular beam epitaxy (OPA‐MBE), varying the Ni content for each sample. 6 nm Ni‐doped α‐${\mathrm{Fe}}_{2}$ $O_{3}$ (111) were prepared as well. The film's crystallinity and structural orientation were monitored by acquiring in situ RHEED patterns during film growth (see Figure S3). After the deposition, samples were annealed in air at 350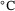 for 23 h. Samples' morphology was studied using AFM (see Figure S4). XPS measurements were carried out using Al Kα emission (1486 eV). Measurements were repeated for selected samples, dried with compressed $N_{2}$ immediately after 30 min of immersion in a 0.1 M NaOH solution, as well as after the *operando* experiments (see Figure S10). XPS data were analyzed with the CasaXPS software [28], estimating the errors of the quantitative analysis with the Monte Carlo method. Probing depths were estimated as 95% information depth attenuation, following the methodology described in ref [29] and calculating the inelastic mean free paths using the SESSA 2.2.2 software based on the NIST database. Photocurrent density $J_{ph}$ and IPCE were investigated with a three‐electrode PEC quartz cell, in a 0.1 M KOH solution, while applying 0.6 V vs Ag/AgCl and illuminating the sample with a solar simulator (1 kW Newport Xenon arc lamp). All $J_{ph}$ were measured under 400 mW· ${\mathrm{cm}}^{-2}$ AM 1.5 G (4 suns) chopped light, subtracting the dark current and normalizing for the electrochemical active area. IPCE data were collected under 1 sun 20 Hz chopped light, measuring the samples' photocurrent as a function of the incident light wavelength between 200 and 700 nm with a 5 nm step. The relative Ni concentration was measured with X‐ray Fluorescence spectroscopy (XRF). Data were collected at the SAMBA beamline using a 10 keV monochromatized beam, maintaining the same distance from the detector (X‐PIPS MirionSDD 13 elements) and acquisition time (10 s). Data from all channels were averaged and corrected for the deadtime. Ni/Fe ratios were estimated from the intensity of the Fe and Ni Kα emission lines corrected for the respective cross sections and optical path absorbance (see Figure S6). Fe and Ni K‐edge XAS spectra were also collected in fluorescence mode at two different angles to avoid artifacts induced by the substrate's diffraction peaks. Several Fe and Ni oxide references were probed in transmission mode. Data were normalized to the incident photon flux and calibrated with reference Fe and Ni foils. *Operando* measurements have been carried out with the cell described in work [30], under a 0.1 M KOH solution, using a 50 μm thick Mylar window to reduce visible light absorbance (see Figure S2). XAS data were collected while in CA mode at different increasing potentials. All applied potentials reported are converted to the reversible hydrogen electrode (RHE). Fixed energy X‐ray absorption photovoltammetry (FEXRAP) data were obtained recording the X‐ray absorption coefficient μ under *operando* conditions for a single incident energy, integrating every 0.05 s for 2 min while illuminating the sample with 2.6 suns chopped light. Measurements were carried out while in static electrolyte to avoid periodic noise introduced by the peristaltic pump. Heating effects were prevented by mounting an infrared filter on the lamp and placing a ventilator in front of the cell. A K‐type thermocouple was also placed above the window, close to the sample. μ was collected synchronously with the light status (monitored with a photodiode in front of the cell), the working current (measured by a Bio‐Logic SP‐300 potentiostat), and temperature. Data were then folded over two chopper periods, and the measurement was repeated and averaged 100 times. A schematic of the experimental setup is shown in Figure S12.

## 3 Structural and Electronic Reconfiguration Upon Ni‐Doping

**FIGURE 1:**
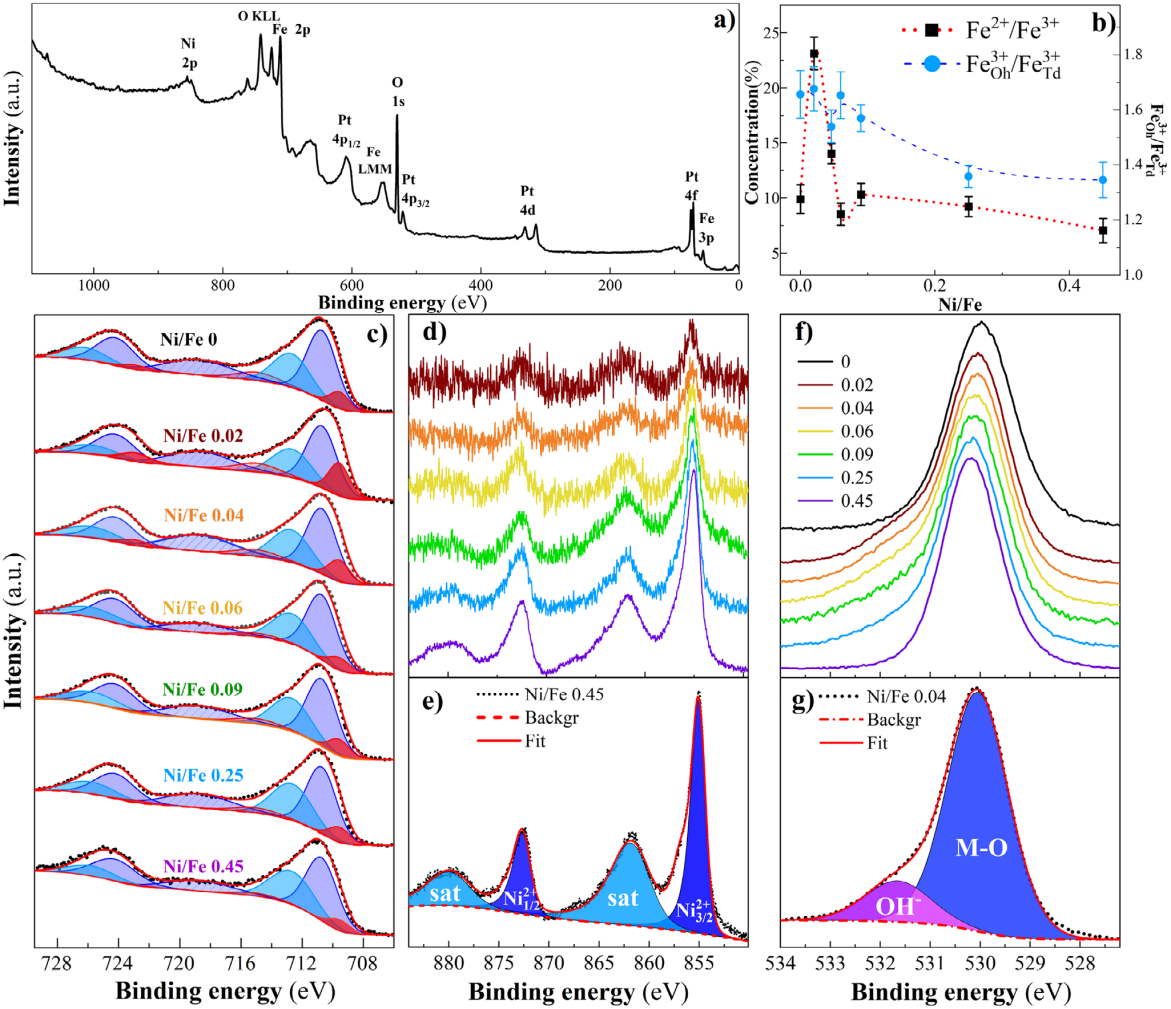
(a) XPS survey; (b) XPS Fe 2p fitting results; (c) Fe 2p spectra (black dots) with fitting curves and Shirley backgrounds (red). ${\mathrm{Fe}}^{2+}$, ${\mathrm{Fe}}_{Oh}^{3+}$ and ${\mathrm{Fe}}_{Td}^{3+}$ components are shown as red, blue and azure areas, respectively; (d) Normalized Ni 2p and (f) O 1s (d) XPS spectra, with relative fits in (e) and (g).

The surface chemical state of the *as‐prepared* samples was studied using XPS. The wide scan spectra exclude the presence of surface contaminants (Figure 1a). The substrate signal can be expected, since following the procedure described in the methods section, we estimate a ∼ 7 nm probing depth for Pt 4f, while only a portion of the film is probed for the Fe and Ni 2p (∼ 4 nm) and O 1s (∼ 5 nm). The Fe 2p spectra (Figure 1b), show for all samples the electronic structure expected for an inverse spinel structure mostly composed of ${\mathrm{Fe}}^{3+}$ species. Following the results of another work on nickel ferrites [31], we estimated the Fe defects concentration at distinct Ni/Fe ratios by fitting the relative ${\mathrm{Fe}}^{2+}$ concentration (more details on fitting protocols and results in the Figure S9). All Fe $2p_{3/2}$ peaks can be decomposed into a ${\mathrm{Fe}}^{2+}$ (709.5 eV), ${\mathrm{Fe}}_{Oh}^{3+}$ (710.7 eV) and a ${\mathrm{Fe}}_{Td}^{3+}$ (712.7 eV) contributions (see Figure 1c) followed by a ${\mathrm{Fe}}^{2+}$ (714.9 eV) and a ${\mathrm{Fe}}^{3+}$ (718.7 eV) satellite peaks. Respective Fe 2$p_{1/2}$ components are found at 722.9 eV (${\mathrm{Fe}}^{2+}$), 724.2 ev (${\mathrm{Fe}}_{Oh}^{3+}$) and 726.2 eV (${\mathrm{Fe}}_{Td}^{3+}$). As shown in Figure 1b, while most samples give a ${\mathrm{Fe}}^{2+}$ concentration within the error margins, we observe a significant increase in the ${\mathrm{Fe}}^{2+}$ content in the 0.02 and, more moderately, in the 0.04 Ni/Fe samples, which hint to the formation of Oxygen vacancies at low dopant concentration. Moreover, we observe a moderate decrease in the ${\mathrm{Fe}}_{Oh}$/${\mathrm{Fe}}_{Td}$ at high Ni concentrations, which suggests a replacement of octahedral Fe sites by Ni. On the other hand, Ni 2p spectra show the presence of ${\mathrm{Ni}}^{2+}$ species (Figure 1f) with $2p_{3/2}$ component at 855 eV and $2p_{1/2}$ at 872.6 eV, the corresponding satellite peaks are found at 861.6 and 879.7 eV. As shown in Figure 1c, no evident changes are revealed as a function of the Ni concentration. Still, the inevitably poor signal from low‐Ni‐concentrated samples hampers quantitative analysis. The O 1s spectra (Figure 1d) exhibit the rise of a component around 1.7 eV above the M‐O lattice oxygen peak (530 eV) in the samples with low and medium concentrations of Ni dopant. Several studies correlated this feature with the presence of oxygen vacancies. Even though this assumption is inadequate since vacant O sites do not emit photoelectrons, the feature can be linked to chemisorbed hydroxyl groups on the film surface, which is favored by the presence of oxygen vacant sites [32, 33], or by a higher binding energy in the presence of the Ni dopant. Since our results evidence abundant hydroxyl adsorption at both low and medium Ni/Fe ratios, ${\mathrm{OH}}^{-}$ concentration appears to be not just related to O defects but also to the presence of Ni ions.

**FIGURE 2:**
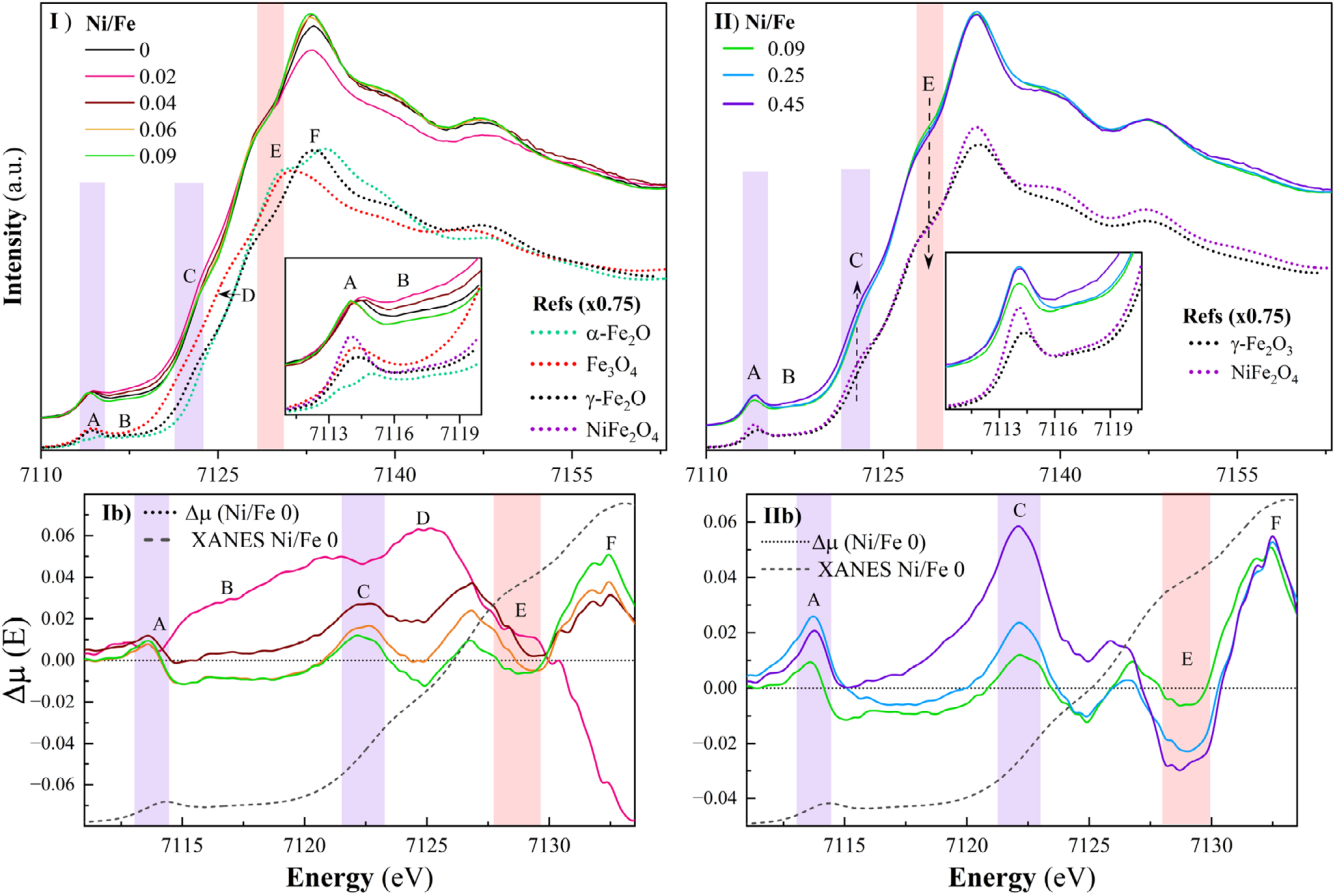
Fe K‐edge XANES spectra of the Ni‐doped γ‐${\mathrm{Fe}}_{2}$ $O_{3}$ samples at low (I) and high (II) Ni concentrations. Reference iron oxide spectra are shown as dashed lines, multiplied by 0.75. Violet bands evidence components attributable to ${\mathrm{Fe}}_{Td}^{3+}$, while red bands highlight features relatable to partially reduced Fe in octahedral coordination [34]. The insets show a magnified pre‐edge region. The spectral differences with respect to the undoped film spectra (black) are shown below (Ib, IIb).

The samples' bulk electronic structure and local coordination were investigated via Fe and Ni K‐edge XAS. Several reference Fe and Ni oxides spectra were also collected to better discriminate the phase formed (see Figure S1). Fe K‐edge results are shown in Figure 2. All samples exhibit a spectrum similar to the γ‐${\mathrm{Fe}}_{2}$ $O_{3}$ reference, with no indication of hematite side product, which confirms the preservation of the cubic spinel structure even at high doping levels. However, modest modulations of several features of the XANES spectra between samples with different Ni content are highlighted by the spectral difference Δμ (Figure 2 Ib, IIb). Compared to the undoped sample (Ni/Fe = 0), at the lowest Ni concentration (Ni/Fe < 0.05) we observe a net decrease of the white line intensity (F, ∼ 7133 eV), higher intensity in region B (7115‐7120 eV), and a moderate redshift of the Fe K‐edge position (D, ∼ 7126 eV). As the Ni content increases from a Ni/Fe ratio of 0.02 to 0.09, the red shift of the edge cancels, the pre‐edge peak (A, ∼ 7114 eV) intensity increases, while feature B and the shoulder E (∼ 7129 eV) recover the intensity of undoped film. On the other hand, the peak C around 7122 eV (C, see Figure 2 Ib) persists from Ni/Fe of 0.04 to 0.09.

The edge position (D) is a well‐known indicator of the Fe average valency, as confirmed by our maghemite (${\mathrm{Fe}}^{3+}$) and magnetite (${\mathrm{Fe}}^{2.67+}$) references [35]. The Fe K pre‐edge (A) corresponds to the Fe 1s→3d electronic transitions. Fe 3d orbitals may hybridize with ligand O 2p orbitals depending on geometry, giving a p character to the final state. The hybridization is possible in tetrahedral geometry and not in octahedral. The intensity then decreases with increasing 3d orbitals occupation. Thus, position and intensity of this feature can be precisely correlated to, respectively, Fe valency and local coordination [36, 37, 38]. Quadrupole transitions allow Fe 1s→3d, but with a coupling around two orders of magnitude weaker compared to dipole transitions. Finally, even in close‐to‐centrosymmetric geometries, this peak can be present (though with very small intensity), since a certain hybridization is possible via thermal and static deviations from the perfect octahedron. There is no precise theoretical description for component B, but the raising of a broad feature above 7115 eV has been recently correlated by X. Sun et al. [39] to the Fe 4p and oxygen 2p orbitals hybridization. The hybridization with the O orbitals can be favored by the presence of O vacancies, which introduce distortions in the ${\mathrm{FeO}}_{6}$ octahedron and were observed to cause B feature raising and white line (F) decrease in similar spinel‐structured Fe oxides [40]. A reduction in the white line intensity (F) can in fact be linked with O vacancy formation as a consequence of a lower density of unoccupied Fe p states. Finally, the two shoulders along the absorption edge (C and E) have been deconvoluted with theoretical XANES simulations by Y. Podkovryina et al. [34] for the magnetite case. Their results suggest that the C feature arises from ${\mathrm{Fe}}_{Td}^{3+}$, while shoulder E is more intense for octahedrally coordinated ${\mathrm{Fe}}^{3+}$ and ${\mathrm{Fe}}^{2+}$.

In agreement with XPS data, these results suggest that Ni doping initially reduces the structural order in the maghemite matrix, possibly by favoring O vacancies formation. The order is then gradually restored at higher Ni concentration. For Ni/Fe above 0.04, the decrease of feature E, the persistence of C, and the rise of A suggest an increase in the relative concentration of ${\mathrm{Fe}}_{Td}^{3+}$. In samples with Ni/Fe > 0.1 (see Figure 2 II, IIb), the pre‐edge (A) intensity increases while its position moderately shifts from 7114.4 to 7114.2 eV, as expected for increased tetrahedral coordinated ${\mathrm{Fe}}^{3+}$ concentration [38]. The decrease of the E feature and the concurrent rise of C confirm the proposed trend. The same modifications are also observed between the maghemite and ${\mathrm{NiFe}}_{2}$ $O_{4}$ references.

**FIGURE 3:**
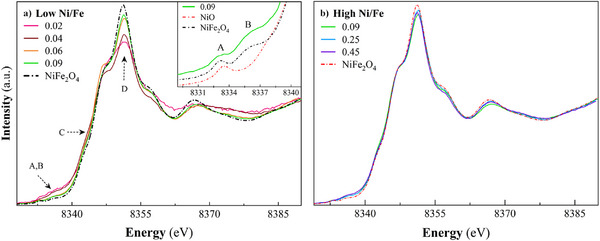
Ni K‐edge XANES spectra for low (A) and high (B) Ni doping levels. Arrows highlight feature modulations across different Ni concentrations. Reference NiO and $\mathrm{NiF}e_{2}O_{4}$ spectra are shown as dashed lines. An inset highlights the pre‐edge region.

A similar modulation is observed at the Ni K‐edge. As the Ni concentration increases, the pre‐edge region (A,B) is hindered, the edge position (C) moderately blueshifts, while the white line (D) significantly rises (see Figure 3I). Comparing our data with several Ni oxide references (see Figure S1), we observed a convergence to ${\mathrm{NiFe}}_{2}$ $O_{4}$ structure at high Ni/Fe, as also pinpointed by the pre‐edge features A and B (Figure 3, inset) appearing for Ni/Fe > 0.06. Feature A can be assigned to the quadrupole allowed 1s→3d transition, while feature B around 8336 eV can be related to dipolar transitions caused by hybridization of the Ni 4p with 3d states of Fe atoms [41]. The modest intensity of peak A supports the octahedral coordination of the Ni ions, where no significant Ni 3d and O 2p hybridization is expected and, hence, no dipole transitions can occur. The EXAFS data confirm the conversion of the sample to the ${\mathrm{NiFe}}_{2}$ $O_{4}$ configuration at high Ni concentration. As shown in the Figure S7, the k‐space Fe K‐edge EXAFS spectra of the sample with no Ni doping perfectly overlap the maghemite reference spectra, while the one with a 0.45 Ni/Fe ratio is in excellent agreement with the ${\mathrm{NiFe}}_{2}$ $O_{4}$ reference. At the Ni K‐edge, the same Ni‐rich sample matches again the ${\mathrm{NiFe}}_{2}$ $O_{4}$ reference spectra. The Ni‐poor sample, on the other hand, shows a signal in phase with the same reference but with a significantly hindered intensity, as expected in the presence of defects.

**FIGURE 4:**
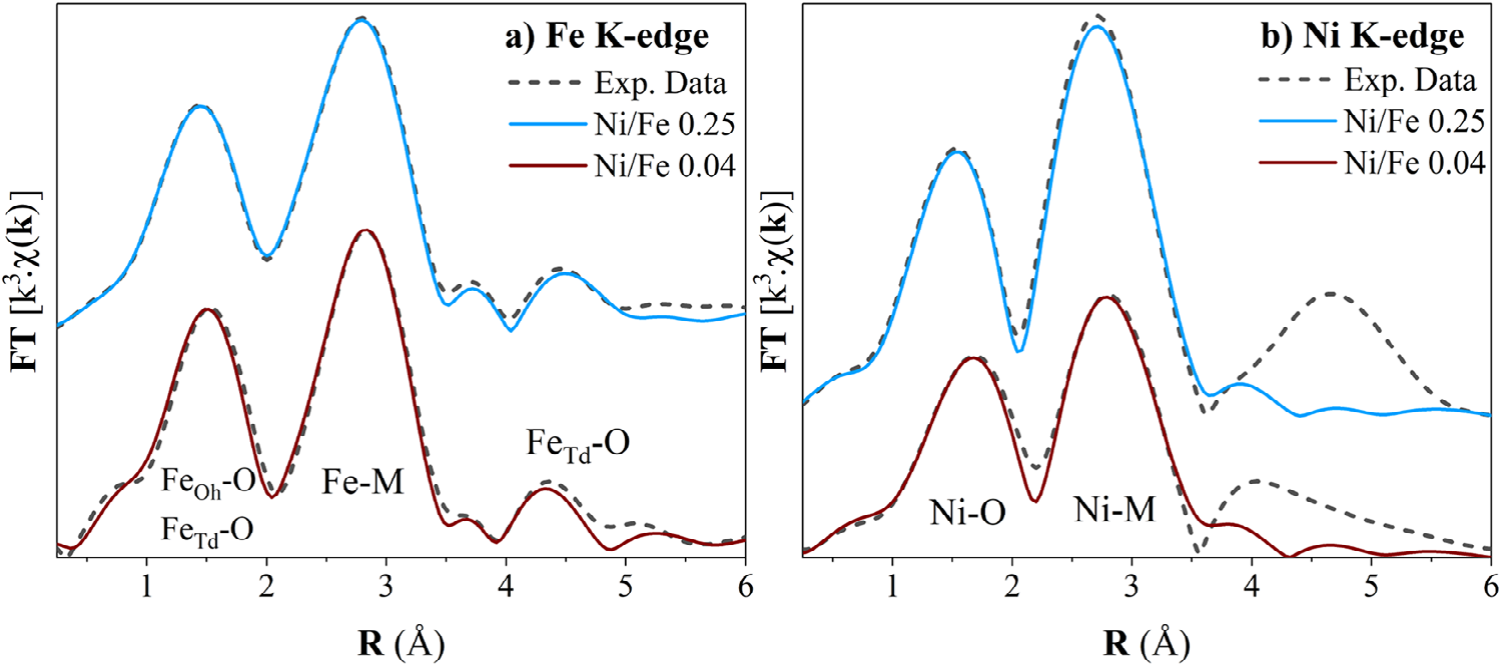
Fits of the Fourier transformed EXAFS spectra at the Fe (a) and Ni (b) K‐edges. Experimental data are shown as dashed lines. *M* stands for Fe or Ni.

To confirm the local structure reconfiguration upon Ni insertion, the Fe and Ni K‐edges EXAFS spectra of selected samples were fitted with the GNXAS package [42]. The initial structure to simulate the two‐body scattering signals was realized by modifying a maghemite structure [43], replacing 1/6th of the Fe octahedral iron with Ni atoms. Spectra were deconvoluted by the two‐body contributions of non‐equivalent M–O and M–M bonds (M being either Fe or Ni), with coordination numbers (CN) fixed to the theoretical values. For the Fe K‐edge, following the results of Conduri et al. [44], the first three shells of ${\mathrm{Fe}}_{Td}$ and ${\mathrm{Fe}}_{Oh}$ atoms were included in the fit with relative distances and $\sigma^{2}$ fitted in a narrow range around their results. The CN of the first Fe‐Ni shell was also fitted to account for the different concentrations of Ni within the two samples. Results are shown in Figure 4, with relative fitting results shown in Table S2. EXAFS spectra and simulations reveal that at the Fe sites (Figure 4a), both samples exhibit a local structure very close to that of pristine maghemite [44] in distances and coordination numbers. However, the Ni/Fe = 0.04 sample shows significantly higher $\sigma^{2}$ in the first Fe‐O shell vs. Ni/Fe = 0.25 ($9\cdot 10^{-3}{Å}^{2}$ vs. $2\cdot 10^{-2}{Å}^{2}$), this can be related to a larger distortion of the oxygen shell in an aliovalent doped and constrained structure. Then, for Ni/Fe = 0.25, the best fit was obtained by increasing the CN in the Fe‐Ni shells and removing one ${\mathrm{Fe}}_{Oh}$ in the ${\mathrm{Fe}}_{Td}$‐${\mathrm{Fe}}_{Oh}$ shell. This is in agreement with a gradual replacement of octahedrally coordinated ${\mathrm{Fe}}^{3+}$ by ${\mathrm{Ni}}^{2+}$ ions. The octahedral coordination of the Ni dopant was confirmed by fitting the Ni K‐edge data (Figure 4b and Table S2). Our EXAFS results show an increase in bond distances and Debye‐Waller factors in the 0.04 Ni/Fe sample. In line with XANES spectra, this correlates with the presence of a distortion of the O shell and or O defects at low Ni concentrations.

The results presented in this section highlighted the structural reconfiguration occurring in the samples as a function of the Ni dopant concentration. The preservation of the maghemite cubic phase upon 3d cation doping (e.g., Co, Ti) was also observed in other two works [45, 46]. Both showed that the dopant ion prefers to occupy the iron octahedral sites, partially filling the Oh vacancies and resulting in minimal lattice distortion. In the case of Ni doping, an analogous mechanism can be expected as a result of the similar ionic radius of octahedrally coordinated ${\mathrm{Fe}}^{3+}$ (0.65 Å) and ${\mathrm{Ni}}^{2+}$ (0.69 Å). Our results confirm that dopant ${\mathrm{Ni}}^{2+}$ ions initially replace octahedrally coordinated ${\mathrm{Fe}}^{3+}$. The charge neutrality is obtained by O vacancy formation for each 2 ${\mathrm{Ni}}^{2+}$/${\mathrm{Fe}}^{3+}$ replacement. However, according to DFT simulations on γ‐${\mathrm{Fe}}_{2}$ $O_{3}$ [47], O vacancy formation that would lead to the reduction of Fe is less favorable compared to an O site where the freed charge can be acquired by other less charged O atoms, which reduce back to the nominal –2 oxidation state. Such intermediately charged $O^{1-}$ anions can be formed by Ni doping as a consequence of the lower valent Ni ions compared to ${\mathrm{Fe}}^{3+}$ ones [48]. Accordingly, our results suggest that as the Ni content increases above 2%, O vacancy formation becomes less likely to occur compared to two $O^{1-}$ states bonded with a ${\mathrm{Ni}}^{2+}$. As the Ni concentration further increases, our results evidence that ${\mathrm{Ni}}^{2+}$ does not only replace ${\mathrm{Fe}}_{Oh}^{3+}$ but can also fill ${\mathrm{Fe}}_{Oh}$ vacancies, promoting O vacancy healing and gradually re‐arranging the structure toward ${\mathrm{NiFe}}_{2}$ $O_{4}$. The transition to the nickel ferrite phase as the Ni/Fe ratio approaches 0.5 is favored, given the similar and thermodynamically stable spinel structure. Both belong to the same space group, only differing in the distribution of Fe and Ni ions, with ${\mathrm{Fe}}^{3+}$ equally distributed between Oh and Td sites in ${\mathrm{NiFe}}_{2}$ $O_{4}$, while ${\mathrm{Ni}}^{2+}$ exclusively occupies Oh sites.

**FIGURE 5:**
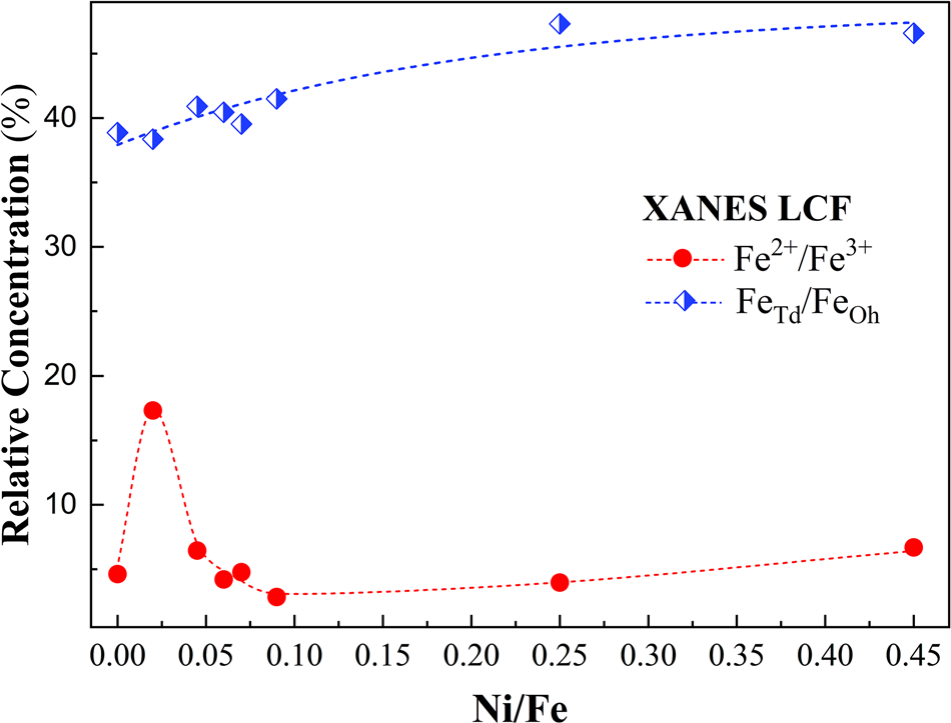
Linear combination fitting results of the Fe K‐edge XANES as a function of the Ni concentration.

The phase formation as a function of the Ni concentration was confirmed via linear combination fitting of the Fe K‐edge spectra with a set of reference Fe oxides collected at the SAMBA beamline. As shown in the Figure S8, both XANES and EXAFS fitting confirm the proposed trend. Three main components are observed: maghemite, ${\mathrm{NiFe}}_{2}$ $O_{4}$, and magnetite, the latter being relatable to the presence of O vacancies that result in ${\mathrm{Fe}}^{3+}$ reduction. In terms of Fe species population, our XANES results (see Figure 5) show an abrupt increase from 5 to 17 % of the ${\mathrm{Fe}}^{2+}$ relative concentration between 0 and 0.02 Ni/Fe ratio, which decreases to 7% at Ni/Fe = 0.04 and then returns below 5% above Ni/Fe = 0.06 (corresponding to O vacancy concentrations of 2.5, 8.5, 3.5 and below 2.5% respectively). On the other hand, the fraction of Fe in tetrahedral sites gradually increases to 50% with the Ni concentration, as a consequence of the conversion to ${\mathrm{NiFe}}_{2}$ $O_{4}$. This is in perfect agreement with our proposed defect formation mechanism, confirming the presence of relevant ${\mathrm{Fe}}^{2+}$ only up to a 2% Ni concentration. In agreement with our results, the maghemite stoichiometry upon ${\mathrm{Ni}}^{2+}$ insertion can be described by the following equation:

(1) $${\left[Fe^{3+}\right]}_{\mathrm{Td}}{\left[Fe_{1-x}^{3-\delta }{□}_{x}Ni_{y}^{2+}\right]}_{2,\mathrm{Oh}}O_{4-z-\delta }^{2-};\;\;\;\;\;\;\left(1+4y+2z\right)=6x$$

With y Ni dopant concentration and δ≃0 for y> 0.02.

## 4 Doping Effect on the EC and PEC Performances

**FIGURE 6:**
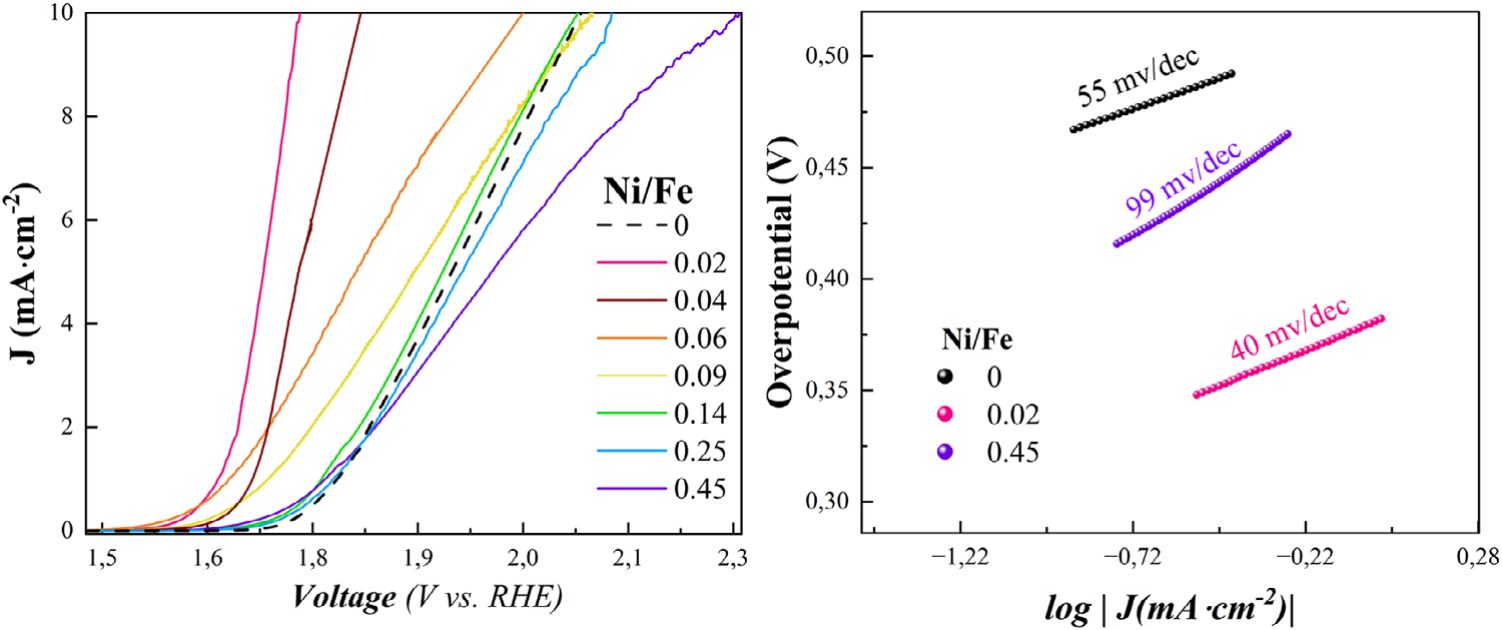
iR‐corrected polarization curves for different Ni concentrations. On the right, Tafel plots of selected samples.

The sample's electrocatalytic activity for alkaline OER was probed by collecting polarization curves via LSV at a 5 mv· $s^{-1}$ scan rate. Results are shown in Figure 6. Compared to the undoped maghemite film, a significant reduction of the OER overpotential is evidenced for doped samples with low Ni content, with a minimal overpotential to reach a current density of 10 mA· ${\mathrm{cm}}^{-1}$ of ∼ 0.5 V for a Ni/Fe ratio of 0.02. On the other hand, the overpotential gradually re‐increases for higher Ni concentrations, exceeding even pristine maghemite for Ni/Fe > 0.1. The Tafel analysis yields a 55 mV/dec slope on the pristine maghemite film, in agreement with previous results on maghemite electrocatalysts [49]. The insertion of a low concentration of Ni not only reduces the reaction overpotential but also facilitates the OER kinetics, as demonstrated by the lower Tafel slope (40 mV/dec). At high Ni concentration, even though the OER onset remains lower than the pristine maghemite case, the reaction kinetics are significantly slower (99 mV/dec at Ni/Fe 0.45).

**FIGURE 7:**
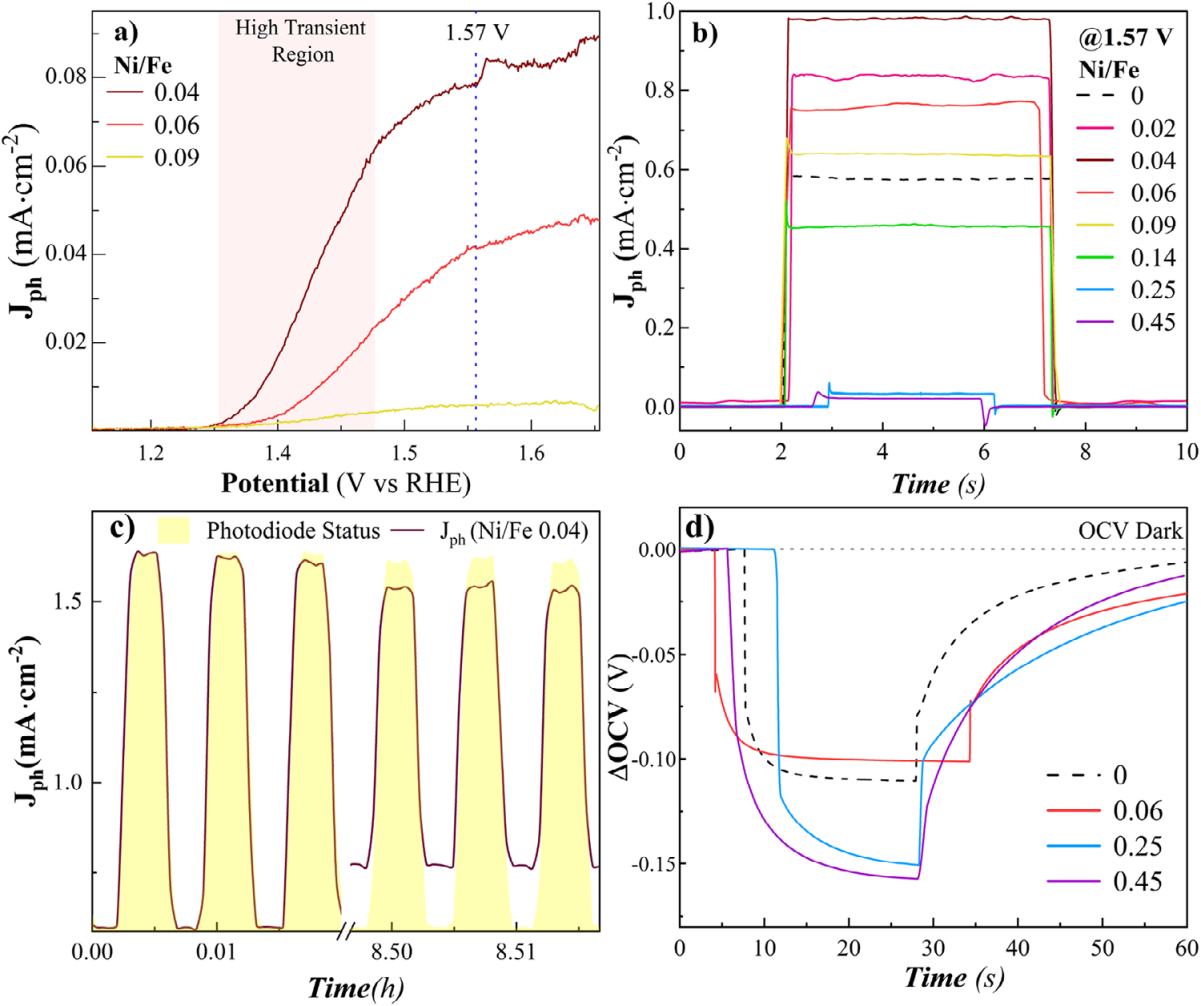
(a) Photocurrent density as a function of the applied potential for three selected samples under 1 sun illumination; (b) Photocurrent density collected at 1.57 V vs RHE under chopped 4 sun illumination on different samples and (c) for an extended time together with the light status. (d) Open circuit potential change under 4 sun illumination.

**FIGURE 8:**
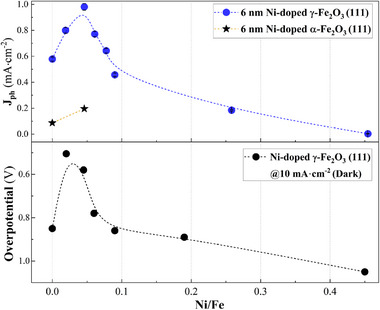
Ni‐doped maghemite (ball) and hematite (star) photocurrent density at 1.57 V under 4 sun illumination as a function of the Ni concentration (top). Below, the relative OER overpotential to reach a current density of 10 mA· ${\mathrm{cm}}^{-1}$ under dark conditions.

The photocurrent density $J_{ph}$ as a function of the applied potential was studied by collecting polarization curves under light exposure and subtracting curves acquired under dark conditions. The results shown in Figure 7a evidence a photocurrent onset around 1.3 V vs. RHE, followed by a low driving force region (red) which is not enough to effectively prevent the recombination of the photo‐generated charges; this results in low photocurrent and relatively high current transients up to 1.46 V. At higher potentials, the photocurrent plateau indicates a region where most photo‐generated charges are efficiently separated and participate to the OER. At these potentials, considering that bulk recombination is unlikely in our ultra‐thin samples, the photocatalysis is governed by the material surface activity [50, 51]. The absolute photocurrent densities were measured at the beginning of this region at 1.57 V vs. RHE. Results are shown in Figure 7b. All samples with low Ni concentration exhibit higher photocurrents and absence of transients, confirming excellent photo‐generated charge separation at this applied potential. Such samples also evidence remarkable photoelectrochemical stability (Figure 8c), with limited attenuation (∼ 24%) of the photocurrent response after more than 8 h of chopped light exposure time. On the other hand, the decrease of the photocurrent intensity accompanied by the reappearance of current transients on the samples with Ni/Fe > 0.1 suggests increased charge‐recombination at high Ni concentrations even in the very low thickness regime (6 nm). Since our XAS analysis showed a ${\mathrm{NiFe}}_{2}$ $O_{4}$‐like structure for the 0.25 and 0.45 Ni/Fe samples, the photocurrent drop might be eventually attributed to an n‐to‐p type conductivity transition, which can occur in ${\mathrm{NiFe}}_{2}$ $O_{4}$ systems [52] or highly doped ${\mathrm{Fe}}_{2}$ $O_{3}$ [53]. Indeed, p‐type conductivity can arise from the hole polaron hopping mechanism, which can be enhanced by the presence of periodic oxygen vacancies or Ni anti‐sites. However, hole hopping occurs between ${\mathrm{Ni}}^{3+}$ and ${\mathrm{Ni}}^{2+}$ while, for low ${\mathrm{Ni}}^{3+}$ concentrations, electron conduction via ${\mathrm{Fe}}^{3+}$‐ ${\mathrm{Fe}}^{2+}$ electron hopping is favored and grants n‐type conductivity [54, 55]. Our analysis did not evidence ${\mathrm{Ni}}^{3+}$ or Ni anti‐site formation, while bulk ${\mathrm{Fe}}^{2+}$ states were observed at low Ni/Fe ratios, thus n‐to‐p transition appears unlikely. To further exclude this possibility, we probed the sample's photovoltage via open circuit measurements under chopped light (see Figure 7d). Our results confirm that both low as well as high Ni/Fe samples evidence a negative photovoltage as pristine maghemite, which is expected for n‐type semiconductors [56, 57] due to an up‐shift of the Fermi level toward the conduction band under illumination.

All photocurrent densities collected under identical illumination and applied potential are shown in Figure 8, together with the relative overpotential estimated under dark conditions. Our results show a volcano trend between the photocurrent and the Ni concentration, with a maximum of ∼ 1 mA· ${\mathrm{cm}}^{-1}$ at a Ni/Fe ratio of 0.04 followed by a gradual decrease at higher Ni content. Such a trend suggests the coexistence of competing phenomena occurring with increasing Ni content. A similar modulation is observed from the OER overpotential, even though in this case the minimum (Ni/Fe 0.02) corresponds to the highest concentration of O vacancies. Figure 8 also shows a comparison between the photocurrent of the investigated maghemite films and of equivalent Ni‐doped hematite films. The beneficial effect of the optimal Ni doping concentration (0.04 Ni/Fe) is confirmed on the hematite sample. However, our results show a ∼5 times higher photocurrent density on the γ‐$Fe_{2}O_{3}$ samples compared to the α‐$Fe_{2}O_{3}$ films.

**FIGURE 9:**
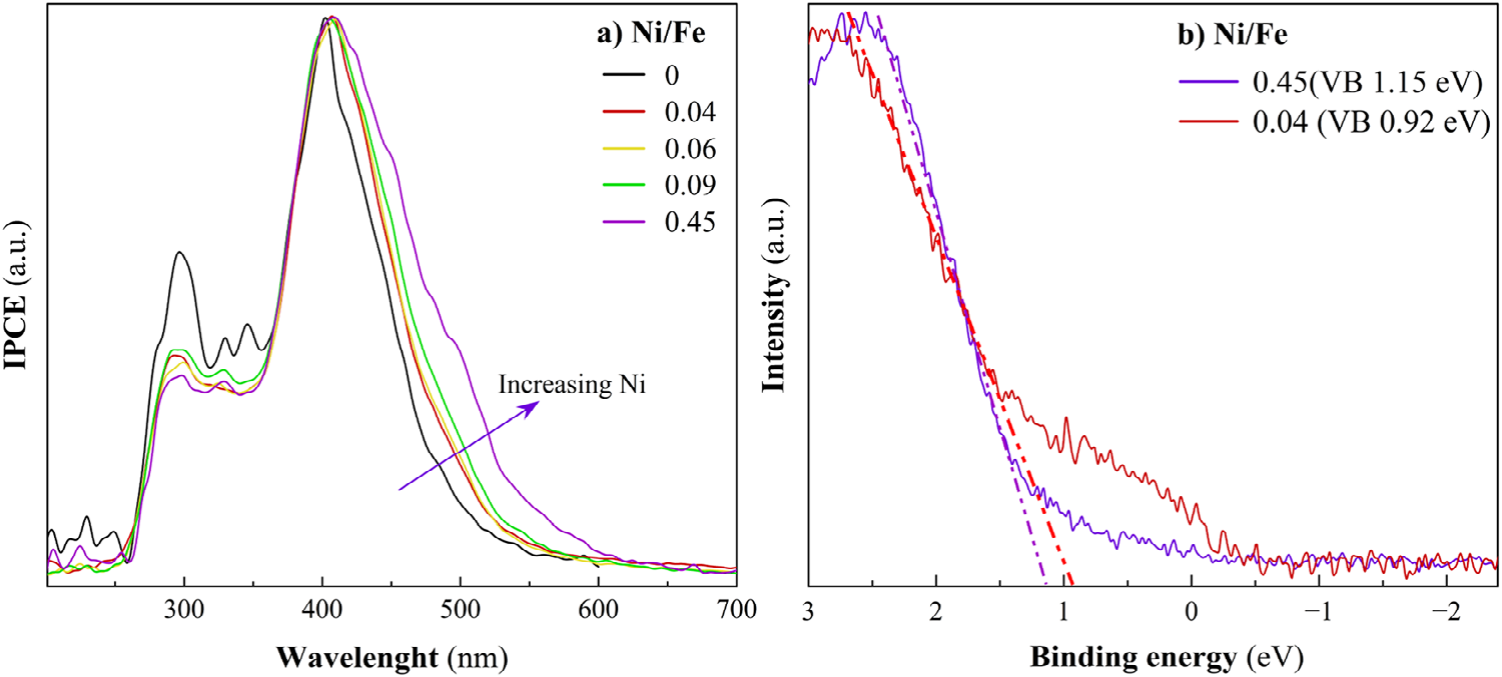
(a) Normalized IPCE curves collected at 1.57 V vs RHE from samples with different Ni content. (b) VB XPS spectrum.

To gain further insight into the band alignment and conversion efficiency of the maghemite photoanodes as a function of the Ni doping, samples were studied via IPCE and VB XPS. The normalized IPCE spectra (Figure 9a) show a common maximum of the photon conversion efficiency around 410 nm and a cutoff wavelength below 600 nm, in agreement with previous works on ${\mathrm{Fe}}_{2}$ $O_{3}$ [14, 58]. As the Ni content increases, the cut‐off wavelength positively shifts, suggesting a decrease of the optical bandgap from 2.29 eV for pristine maghemite to 2.03 eV for a 0.45 Ni/Fe ratio. Our VB spectra are shown in Figure 9b. The Ni ferrites VB is composed of 2p O and 3d Ni and Fe states. In agreement with a previous work on defective ${\mathrm{NiFe}}_{2}$ $O_{4}$ [59], at 0.45 Ni/Fe we observe a lower energetic feature at the top of the VB, which can be ascribed to Ni states. In the 0.04 Ni/Fe case, the raising of a feature around 1 eV can be ascribed to defect states induced by the presence of O vacancies, though a stronger Pt substrate contribution cannot be excluded. On the other hand, our fit highlights a positive shift of the VB maximum at higher Ni concentrations. Considering that our XAS results confirmed a reordering of the structure at high Ni content, an upward shift of the VB might occur upon Ni insertion due to Ni 3d/O 2p hybridization [60]. In agreement with our IPCE data, the VB shift narrows the bandgap, improving visible light photon‐to‐current conversion in the visible range.

### 4.1 Catalyst Reconfiguration Under *Operando* Conditions

**FIGURE 10:**
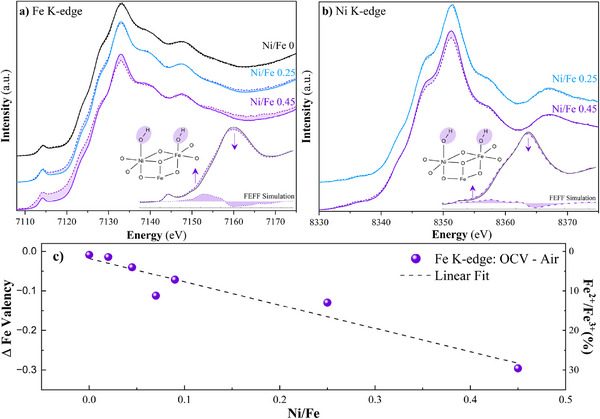
Fe (a) and Ni (b) K‐edges XANES spectra from selected samples with distinct Ni/Fe ratio, collected before (continuous curves) and after (dashed) electrolyte insertion. Colored areas highlight spectral modifications. In the insets, the respective calculated spectra for stoichiometric and hydroxylated (dashed) maghemite, with ${\mathrm{OH}}^{-}$ bonded at the ${\mathrm{Fe}}_{Oh}$ and Ni sites as in the sketches; (c) Variation of the Fe valency upon electrolyte insertion estimated from the Fe K‐edge shift.

The samples' electronic reconfiguration under *operando* conditions was studied by probing the Fe and Ni K‐edges under a 0.1 M KOH solution while in CA mode. First, XAS spectra were collected before and after electrolyte insertion, waiting for open circuit potential (OCV) stabilization to probe the electrode/electrolyte equilibrium state. XANES spectra at both edges are shown in Figure 10a,b. As a general trend, we notice a white line decrease and an increased pre‐edge intensity at both Fe and Ni K‐edges upon electrolyte insertion. In the Fe K‐edge case, these modulations also result in a moderate redshift of the edge position, suggesting a partial reduction occurring at the Fe sites, which might be related to hydroxylation.

To confirm this assertion and identify the absorption sites, the theoretical modulations induced by ${\mathrm{OH}}^{-}$ absorption on the Fe and Ni K‐edge spectra were investigated via multiple‐scattering calculations using FEFF 8.4 [61] using the modified maghemite structure used for the EXAFS analysis. After initial refining with a small 2.4 Å cluster only containing the first O coordination shell (see Figure S16), actual XANES simulation was carried out with a 4 Å cluster around the two distinct Fe sites, which included the first four coordination shells around the photoabsorber. The hydroxyl absorption was simulated by bonding an H atom to an O atom in a typical hydroxyl bond, fixing 0.96 Å the O‐H length along a 28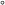 tilted direction compared to the Fe–O direction, also elongated by 0.02 Å to account for the expected Fe–O bond weakening [62, 63, 64]. For absorption at the ${\mathrm{Fe}}_{Td}$ sites, our simulations predict attenuation of the pre‐edge peak intensity that is not observed in our experimental data (see Figure S16). Hence, ${\mathrm{OH}}^{-}$ bonds were coordinated only at the octahedral iron sites on the oxygen displaced along the x crystallographic direction. The final Fe K‐edge XANES was then obtained as a weighted average of the ${\mathrm{Fe}}_{Td}$ and ${\mathrm{Fe}}_{Oh}$ contributions according to the theoretical site distribution. Our results confirm that hydroxyl absorption provokes modulations similar to those in our experiments (see inset in Figure 10a), perfectly matchingthe experimental results up to Ni/Fe 0.25. At the highest Ni concentration (Ni/Fe 0.45), a relatively higher increase of the Fe K pre‐edge peak background is observed. At such Ni concentrations, compared to the modified maghemite structure used for the simulation, almost every Fe has a Ni neighbor, and hence each absorbed ${\mathrm{OH}}^{-}$ transfers charge to Ni that pushes it to higher valent adjacent Fe, resulting in the valence change evidenced in Figure 10c and in a sort of increasing background effect between the pre‐edge peak and the edge. The Ni K‐edge spectra were simulated with the same approach. As shown in Figure S17, while adding the ${\mathrm{OH}}^{-}$ bond only with ${\mathrm{Fe}}_{Oh}$ results in a modest redshift of the Ni edge, a white line hindrance is achieved by adding an identical ${\mathrm{OH}}^{-}$ bond at both octahedral Fe and Ni sites (see inset in Figure 10b). Hence, the higher modulations experimentally observed on Ni‐rich samples can be related to a more abundant hydroxyl absorption in the presence of Ni. The ${\mathrm{OH}}^{-}$ coverage can be related to the fraction of ${\mathrm{Fe}}^{2+}$ species formed upon electrolyte insertion, which can be calculated from the shift of the Fe K‐edge at half the absorption jump [35]. Using the ∼ 0.29 oxidation state unit/eV calibration factor obtained considering the edge positions of a magnetite (${\mathrm{Fe}}^{2.67+}$) and maghemite (${\mathrm{Fe}}^{3+}$) references, we obtained a linear dependence between ${\mathrm{OH}}^{-}$ concentration and Ni/Fe ratio (see Figure 10c), which confirms a facilitated ${\mathrm{OH}}^{-}$ absorption in the presence of Ni sites. This assertion is further confirmed by XPS measurements (see Figure S10) that evidence a higher adsorbed ${\mathrm{OH}}^{-}$ concentration after 40 min immersion in a pH 13 electrolyte in Ni‐irich samples compared to a Ni‐poor one that underwent the same treatment.

**FIGURE 11:**
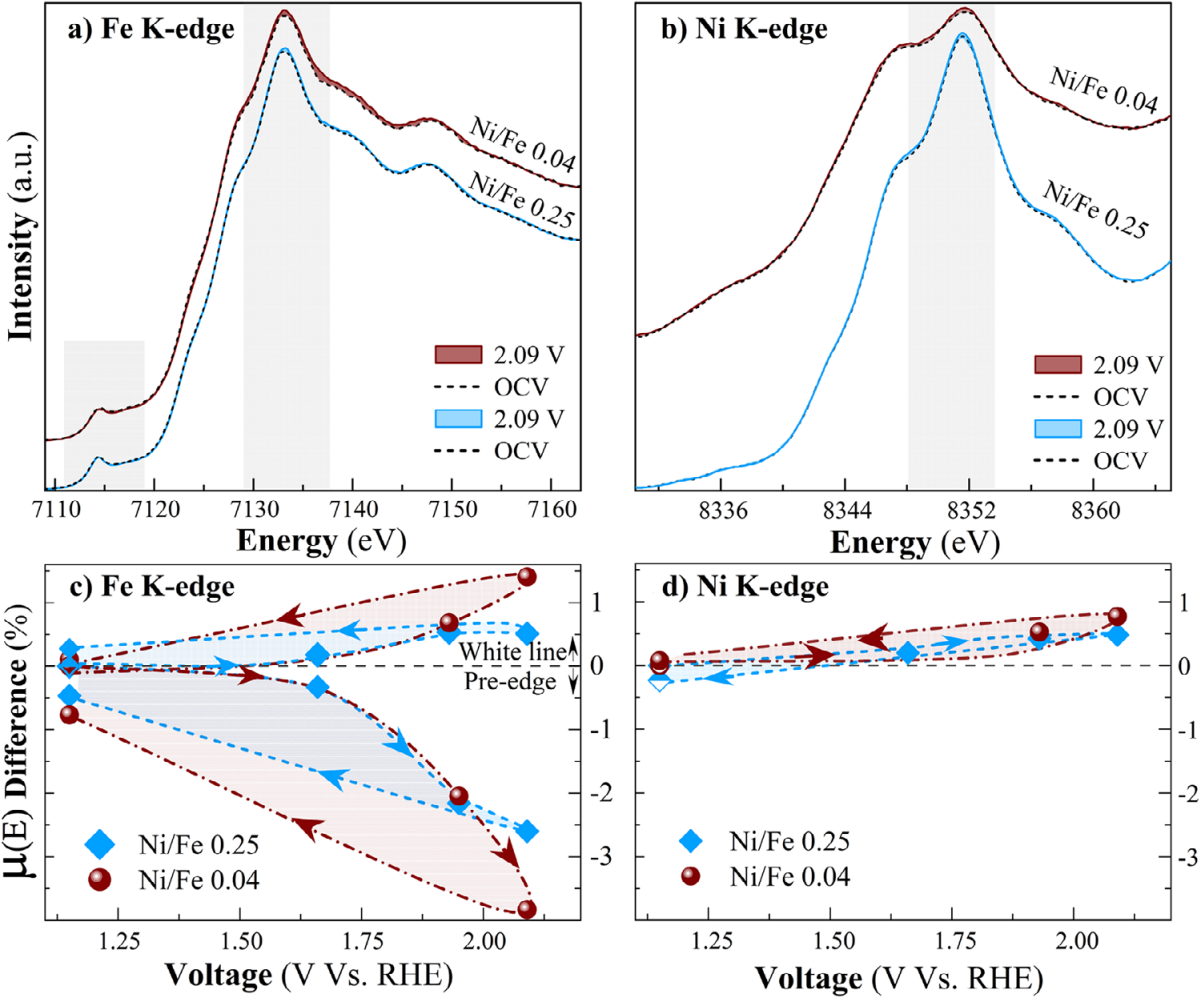
Fe (a) and Ni (b) K‐edges XANES spectra collected from two selected samples at different applied potentials under 0.1 M KOH. Colored areas highlight spectral changes at higher potentials. Grey bands evidence the integration regions. (c,d) Integral of the spectral difference as a function of the applied potential for the optimal (brown) and sub‐optimal (azure) sample.

Fe K‐edge and Ni K‐edge spectra were collected at rising and selected potentials. Results for two selected Ni/Fe ratios are shown in Figure 11. Any clear trend is evidenced in the EXAFS spectra over the whole range. However, a modest potential‐dependent modulation is observed in the XANES: white line intensity increases and the pre‐edge features decrease with increasing applied potential. At the Fe K pre‐edge, the higher modulation occurs at the so‐called B feature (as in Figure 2a) few eV above the pre‐edge peak. The spectral difference Δμ helps in quantifying the trend (see Figure S19): at the Fe and Ni K‐edge white line and the Fe K pre‐edge, we integrate the spectra in a ± 5 eV range around the investigated features. At Fe K‐edge, both samples evidence a modest increase of the white line and hindering of the pre‐edge region intensities above the OER onset (1.6 V vs RHE, Fig. 11c). A similar, more contained white line trend is observed at the Ni K‐edge (Fig. 11d). These modulations might be attributed to the oxidation at the ${\mathrm{Fe}}_{Oh}$ and Ni active sites that had adsorbed ${\mathrm{OH}}^{-}$, forming the $O^{*}$ and ${\mathrm{OOH}}^{*}$ reaction intermediates upon ${\mathrm{OH}}^{-}$ deprotonation and O–O bond formation; this is usually considered the rate‐determining step in OER catalysis on ${\mathrm{Fe}}_{2}$ $O_{3}$ [65]. When the potential is decreased back to OCV conditions, the pre‐edge and white line intensities are partially restored to the initial values due to the metastability of the reaction intermediates and the re‐hydroxylation of the sample's surface. Our analysis evidences a higher Δμ(%) modulation in the optimal 0.04 Ni/Fe sample, particularly at the Fe sites (∼ 1.5% increase at the Fe white line and 3.8% decrease in the pre‐edge), while minor changes are observed at the Ni sites (∼ 0.8% white line increase and no pre‐edge variation).

### 4.2 FEXRAP ‐ Surface Species Kinetics Under Chopped Light

**FIGURE 12:**
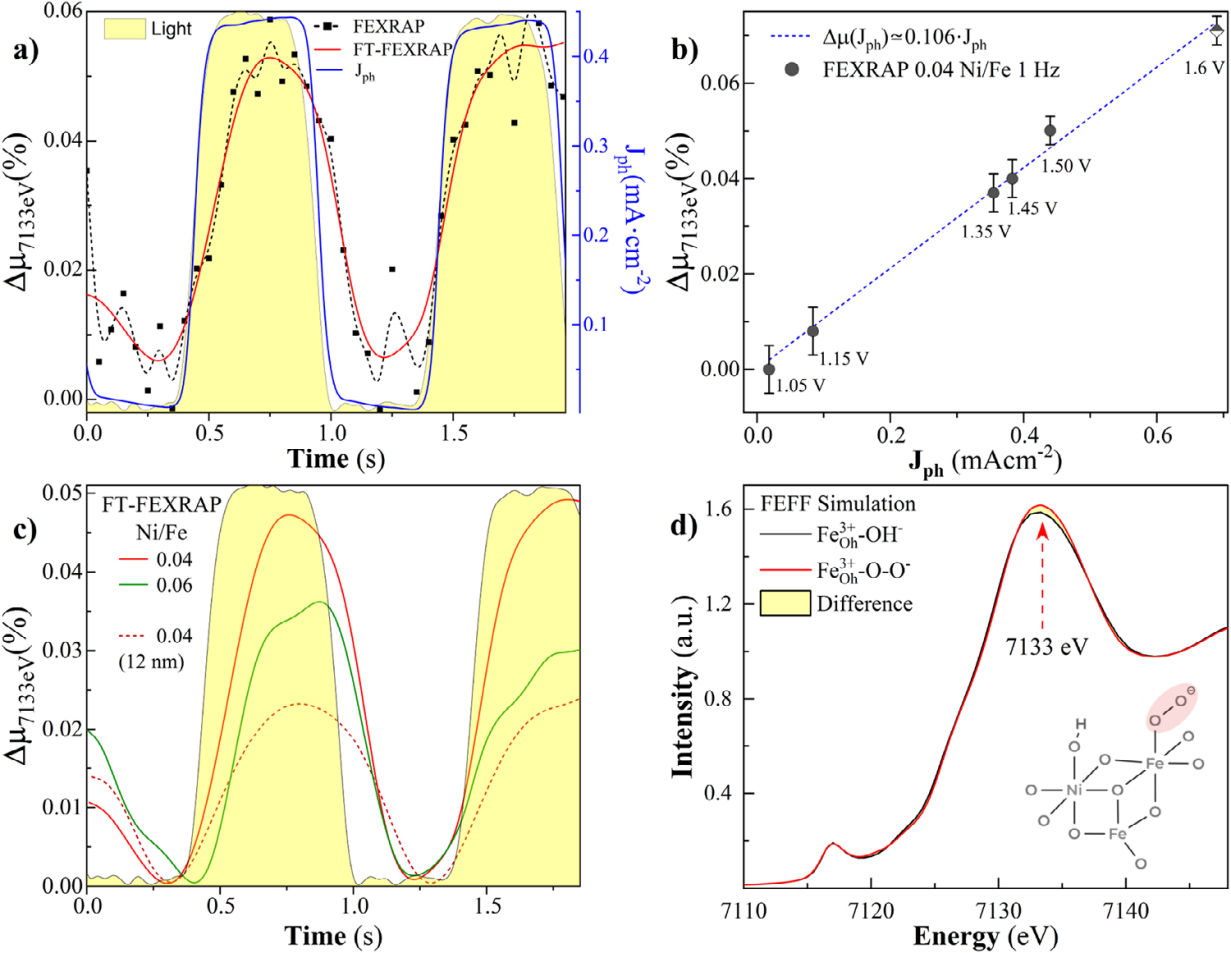
(a) FEXRAP measurement at the Fe K white line on the 0.04 Ni/Fe sample under 1 Hz chopped illumination. The yellow area indicates illumination. Normalized X‐ray fluorescence intensity data are shown as black squares, while the red curve indicates the Fourier smoothed signal. The relative photocurrent is shown as a blue curve; (b) FEXRAP intensity on the 0.04 Ni/Fe sample while at different applied potentials and identical 1 Hz illumination. The fitted linear relation between FEXRAP intensity and photocurrent density is shown as a blue dashed line; (c) Comparison with the Fourier smoothed FEXRAP signals collected from a 12 nm thick 0.04 Ni/Fe sample (red dashed), a 6 nm 0.06 Ni/Fe sample (green) and the optimal 6 nm 0.04 Ni/Fe sample (red); (d) Calculated Fe K‐edge spectra of the Ni‐doped maghemite in the presence of hydroxyl bonds (black) and superoxo bond at the ${\mathrm{Fe}}_{Oh}$ sites (red).

To study the dynamics occurring in the samples during PEC OER, the *operando* XAS experiments described in the previous paragraph were repeated while illuminating the sample with the solar simulator. At 1.57 V, we observed an extremely small increase in the Fe K‐edge white line intensity upon illumination (see Figure S11). However, the relative variation is in many samples close to the noise level, thus not clearly reproducible. To isolate the effect of illumination and enhance the signal‐to‐noise ratio, we applied the FEXRAP technique as described in the experimental section. Figure 12a shows the FEXRAP results collected by probing the Fe K‐edge white line (7133 eV) from the sample with the best PEC performances (Ni/Fe = 0.04) while applying a 1.57 V under 2.6 suns equivalent 1 Hz chopped illumination. Data are shown as % variation of the absorption coefficient, which is obtained by the normalized fluorescence intensity modulation. Our results evidence a reversible increase in the white line intensity under illumination. The effect is indeed extremely small (up to 0.1 %, see also Figure S15), below the sensitivity of a conventional XAS experiment. To exclude the possibility of thermal/mechanical induced artifacts, the experiment was repeated at other incident energies above the Fe K‐edge, and the fluorescence data were compared with the simultaneously measured sample temperature. As shown in Figure S13, the maximum temperature increase is negligible (< 0.3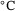) and the FEXRAP modulation disappears at different incident energies.

The experiment was repeated on the same sample, varying the applied potential. As shown in the Figure S14, the poorer signals at lower applied potentials complicate an accurate visualization and quantification of the signal intensity. The signals were smoothed (FT‐FEXRAP) and the maximum Δμ(%) under illumination was measured with a relative error equal to the noise fluctuation. As shown in Figure 12b, our results evidence a linear dependence of the Δμ(%) at 7133 eV with the photocurrent density synchronously measured, linking the FEXRAP detected signal to the PEC activity. To further confirm this relation, the experiment was carried out on other samples under identical conditions. As shown in Figure 12c, the sub‐optimal sample with a 0.06 Ni/Fe ratio shows lower relative variation of the white line intensity. No clear modulation was recoverable from even less efficient samples. The same figure also suggests a surface‐related nature of this effect, since repeating the experiment on a thicker (12 nm) Ni/Fe sample with the optimal doping level (0.04 Ni/Fe) resulted in a halving of the FEXRAP modulation despite the higher photocurrent density (see Figure S5).

The observed reversible increase of the white line intensity under light illumination might be related to a light‐induced increase of the concentration of unoccupied Fe p states, which can be expected to scale with the photocurrent density as a consequence of a higher density of photogenerated holes accumulating on the sample surface. Our time‐dependent data, however, shows a relatively slow rise and decay of the white line intensity modulation compared to the sample photocurrent. Fitting the rise and decay time of our data to standard exponential functions (see Figure S15), we estimated characteristic timescales within the range of τ = 350 ± 50 ms for the white line modulation of all probed samples, and a shorter decay time for the photocurrent (∼ 46 ms). The obtained photocurrent's τ is consistent with previous works on ${\mathrm{Fe}}_{2}$ $O_{3}$ [66], where the authors estimated 20 ms for external circuit electron collection following the more rapid (μs) electron/hole recombination phase, which, however, becomes slower as the applied potential becomes more anodic. Conversely, the longer τ of the FEXRAP signal excludes that we are directly observing the hole photogeneration (∼ 10 ps [67, 68]) recombination, or even the bulk holes migration toward the sample surface, which can be expected to occur within a ns timescale. This suggests that we are actually probing a surface chemical process. A first option might be a reduction in absorbed hydroxyl concentration under illumination, as a consequence of a local pH decrease, which can occur during photocurrent flowing. However, the expected decrease in the pre‐edge region was not evidenced by our FEXRAP approach, and the local pH stability in our highly alkaline solution for relatively low current densities (< 1 mA· ${\mathrm{cm}}^{-1}$) is consistent with the results of other works [69, 70]. Recent works on α‐${\mathrm{Fe}}_{2}$ $O_{3}$ photoanodes [71, 72, 73] highlighted longer decay time (ms to s) for the light‐driven surface species compared to the photocurrent. Such species result from the oxidation of ${\mathrm{OH}}^{-}$ chemisorbed at the Fe site upon hole injection, i.e., they can be responsible for the observed increase of the density of unoccupied Fe p states on the surface active sites. The theoretical XANES modulation upon formation of OER intermediates was simulated with the FEFF 8.4 software, starting from the hydroxylated structure used in Figure 10a. Our results show that the replacement of a first shell and a second shell hydroxyl bonds at the ${\mathrm{Fe}}_{Oh}$ sites with two Fe–O–O bonds results in a significant increase of the whiteline intensity with minor effects on the rest of the XANES spectra (see Figure S18). The bond lengths and structures were refined by varying the Fe–O–O angle and the Fe–O and O–O distances within the theoretical values expected for iron‐superoxo complexes [64, 74, 75]. The best result that minimizes the spectral variations except for the white line region is shown in Figure 12d, and was obtained for a 0.05 Å elongation of the Fe–O bond along the x direction and a 1.39 Å O–O distance along a 123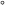 tilted direction.

## 5 Discussions

Two OER pathways are generally proposed, depending on the sample structure and superficial terminations: the adsorbate evolution mechanism (AEM) or the lattice‐oxygen‐mediated mechanism (LOM). In an alkaline medium, the AEM starts with the chemisorption of ${\mathrm{OH}}^{-}$ in the active site and deprotonates by interacting with another hydroxyl ion. The resulting $O^{*}$ surface adsorbed intermediates bond again with ${\mathrm{OH}}^{-}$, forming ${\mathrm{OOH}}^{*}$, which finally desorbs into $O_{2}$, leaving a vacant site that will be occupied by another ${\mathrm{OH}}^{-}$. The main scaling factors of a catalyst following this pathway are the ratio of the produced intermediates and their adsorption strength, which should not be too high or too low. In the LOM path, the ${\mathrm{OOH}}^{*}$ intermediate is formed by activating a lattice oxygen with electronic states near the Fermi level, directly undergoing the ${\mathrm{OH}}^{-}$ nucleophilic attack. The minimal structural and electronic changes under the OER regime evidenced in our samples support an AEM path for the reaction over the LOM. Our results evidence the concurrence of several effects induced by Ni doping on the AEM kinetics. First, the ${\mathrm{OH}}^{-}$ binding energy increases as a function of Ni concentration, resulting in the decrease of the OER onset potential as a consequence of a facilitated hydroxyl absorption. An optimization of the hydroxyl absorption at the ${\mathrm{Fe}}_{Oh}$ sites by Ni doping can be expected, considering that theoretical works individuate ${\mathrm{Fe}}_{Oh}$ as preferred sites for hydroxyl stable absorption and consequent protonation in ${\mathrm{NiFe}}_{2}$ $O_{4}$ [76], and that the interplay between ${\mathrm{Fe}}^{3+}$ and ${\mathrm{Ni}}^{2+}$ sites was shown to modulate reactants absorption by varying the distance between the metal sites d band center and the Fermi level [77, 78]. On the other hand, our analyses show significantly lower OER overpotentials in the low Ni‐containing sample, with a gradual worsening of the OER reaction rates for Ni/Fe ratios above 0.06. Considering that the minimal overpotential corresponds to the maximum of O vacancies concentration, the modulation of the electrocatalytic performances can be related to the bulk conductivity increase and charge transfer facilitation in the presence of O vacancies, which are known to reduce energy barrier of reaction surface intermediates formation for both α‐${\mathrm{Fe}}_{2}$ $O_{3}$ as well as Ni ferrites phases [65, 79, 80]. Above Ni/Fe = 0.06, where the O defects have been healed, the stronger ${\mathrm{OH}}^{-}$ binding energy and the gradual filling of Fe vacancies by ${\mathrm{Ni}}^{2+}$ further hinder the material's performance. In fact, DFT analysis on γ‐${\mathrm{Fe}}_{2}$ $O_{3}$ shows that Fe vacancies in the proximity of the surface not only increase the material's conductivity but can also facilitate surface oxidation reactions, reducing the electronic charge on superficial O atoms [47]. Our XANES analysis under *operando* conditions confirms the facilitated surface oxidation of intermediate species on low Ni‐containing samples. Moreover, the larger spectral modulation observed at the Fe K‐edge suggests a higher degree of ${\mathrm{OH}}^{-}$ deprotonation and subsequent $OOH^{*}$ formation on species chemisorbed on Fe sites, confirming the ${\mathrm{Fe}}_{Oh}$ sites as the active ones for the OER [76]. Considering that O and ${\mathrm{Fe}}_{Oh}$ are the most stable surface terminations for (111) maghemite on Pt (001) [24] and the gradual reduction of ${\mathrm{Fe}}_{Oh}$ fraction with Ni concentration, a decrease in active site density is also expected at high Ni concentration.

The photocurrent density as a function of the Ni/Fe ratio follows a similar trend to the OER overpotential, suggesting that the PEC performance is mainly influenced by the optimization of the surface catalytic activity. However, the shift of the optimal Ni/Fe ratio to 0.04 suggests the presence of additional effects. Similar optimal doping levels were reported for transition metal dopants on epitaxially grown α‐${\mathrm{Fe}}_{2}$ $O_{3}$ films [14, 15, 81], while a moderate bulk O vacancy concentration was also observed to increase the $J_{ph}$ of these systems [82]. In agreement with these works, we also observe an increase in the recombination rates for Ni/Fe > 0.1, which our analyses link to poorer hole mobility upon O and Fe vacancies filling and reduction of ${\mathrm{Fe}}_{Oh}$ active site density. The result is the observed current transients assignable to charge trapping of the photogenerated charges as small polarons [67]. On the other hand, our analyses show that Ni doping improves the photon‐to‐current conversion efficiency in the visible range as a consequence of a moderate, gradual upshift of the VB maximum and a decrease of the bandgap. This band‐tuning mechanism is consistent with the n‐type doping expected from Ni insertion [77, 83] and, combined with the O‐vacancy‐induced boosting of surface kinetics, shapes the volcano trend highlighted by our data. Our FEXRAP measurement reveals a light‐driven, reversible surface‐bound increase in the density of unoccupied p states at the Fe site. Such modulation is linearly dependent on the photocurrent and has a characteristic rising and decay time of ∼ 300 ms. The PEC‐OER on ${\mathrm{Fe}}_{2}$ $O_{3}$ is well defined as a sequence of proton‐coupled charge transfer steps [71, 84]:

(2) $$\begin{array}{c} h\nu \to h^{+}+e^{-}\to nkT \end{array}$$

(3) $$*OH^{-}+OH^{-}+h^{+}\to *O^{-}+H_{2}O$$

(4) $$*O^{-}+OH^{-}+h^{+}\to *\mathrm{OO}H^{-}$$

(5) $$*\mathrm{OO}H^{-}+OH^{-}+h^{+}\to *OO^{-}+H_{2}O$$

(6) $$*OO^{-}+OH^{-}+h^{+}\to *OH^{-}+O_{2}$$

The reaction starts with photon absorption (Equation 2) that cause $e^{-}$/$h^{+}$ photogeneration. The holes migrate toward the electrolyte and can suffer recombination in so‐called trap states. The holes that do not recombine can oxidize surface F $Fe^{3+}$ sites that had absorbed hydroxyl, forming highly valent Fe = O species (Equation 3), which is considered to be the pre‐equilibrium step [71] before the rate‐determining step of the O–O bond formation (Equation 4) passing through the 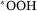 intermediate. The $O_{2}$ release is finally accomplished by two other fast hole injection and hydroxyl bonding reactions (Equations 5 and 6). In the case of hematite in highly alkaline media, when the surface hole concentration is sufficient to form three $*O^{-1}$ in proximity, direct superoxo formation without the hydroperoxo step is thermodynamically favored due to a lower formation energy of the O–O bond in the following reaction [84]:

(7)

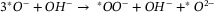

Our FEXRAP signals can be linked to the oxidation of the absorbed hydroxyl to $*O^{-1}$ surface species (Equation 3), which is followed by the superoxo formation upon O–O bond formation. The capability of single‐energy X‐ray absorption has been widely exploited to follow the kinetics of chemical reactions during voltammetries (so‐called FEXRAV [85]) or light pulses on metal oxides photoanodes [86]. The observed characteristic accumulation times are consistent with surface species formed during the rate‐determining step [84, 87]. Indeed, in recent experimental works on hematite thin films [71, 72] the authors observed a longer lifetime (∼ 100 ms) of the light‐induced surface species compared to the photocurrent, in excellent agreement with our results. However, such long‐lived species were assigned to ${\mathrm{Fe}}^{4+}$. In this case, the long‐lived hole accumulation resulting in the emptying of 3d orbitals would give rise to Fe K‐edge position shift and significant changes in the pre‐edge region, which were never detected in our experiments. On the other hand, our results suggest that on Ni‐doped maghemite, long‐lived superoxo intermediates form at hydroxylated ${\mathrm{Fe}}_{Oh}$, within the proximity of the electrolyte interface and in a concentration depending on the density of surface holes. Such species, in agreement with our multiple‐scattering simulations, weaken the Fe–O bond and increase the density of unoccupied p states, leading to no significant XANES changes except an increase in the Fe K‐edge white line intensity. In support of this surprising result, in the work of Li et al., [88], the authors observed that the rate‐determining step and the 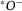 and iron‐peroxo species lifetime depend on the pH, potential and light intensity. In our experimental conditions, long‐lived iron‐peroxo species originating from short‐lived 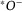 bonded to $Fe^{4+}$ can be expected. Moreover, considering a recent and rare free energy diagram of the AEM of ${\mathrm{NiFe}}_{2}$ $O_{4}$ [76], ∗O−O species are expected to be relatively metastable. Hence, a long decay time of metastable superoxo/hydroperoxo species may also be expected. The FEXRAP signals are hence consistent O–O bond formation at the ${\mathrm{Fe}}_{Oh}$ sites upon surface absorbed ${\mathrm{OH}}^{-}$ oxidation. Such metastable superoxo species formation can be proposed to precede the rate‐determining steps on γ‐$Fe_{2}O_{3}$, bypassing the hydroxo step, due to the superior surface reaction kinetics [89] and long hole lifetimes [22] of the spinel phase, leading to the observed 5 times higher photocurrent density compared to the hematite phase. Around a 0.04 Ni/Fe concentration, the solar‐to‐current conversion efficiency and surface activity are optimized, leading to higher surface hole density under illumination and, hence, superoxo concentration. On the other hand, as both O and ${\mathrm{Fe}}_{Oh}$ vacancies are healed at high Ni content, the conductivity drops and so does the surface hole density, preventing direct superoxo formation. The magnitude of the FEXRAP signal (∼0.1%) observed in the 0.04 Ni/Fe sample is consistent with the observation of the described effect. In fact, considering the $d_{111}$ spacing between maghemite planes (∼ 4.8 Å), around 13 atomic planes are contained in a 6 nm thin film. In a flat surface approximation and assuming that all octahedral Fe sites are active, if O–O bond formation is limited to the surface layer, the theoretical white line increase (2% in our FEFF simulation) should be divided by 13. Hence, a ∼ 0.16% modulation should be expected.

## 6 Conclusions

In this work, we investigated the structural and electronic reconfiguration induced by Ni doping on maghemite (111) thin films, and their effect on the material performances for solar‐driven OER. Our results show a significant improvement of the PEC performances at low Ni/Fe ratios, with an optimal value around 0.04. We demonstrated how such a trend is due to the optimization of the O and Ni defect concentration and site distribution. We revealed an increase in the hydroxyl binding energy and a decrease in the material's bandgap with increasing Ni concentration. Concurrently, Ni ions initially replace ${\mathrm{Fe}}_{Oh}^{3+}$ and form O vacancies, which improves the surface catalytic reactivity. As the Ni content increases, however, both O and octahedral Fe vacancies are gradually healed, reducing the material's conductivity (i.e. holes diffusion length) and the surface species stabilization. Our results also confirm that maghemite‐based photoanodes can significantly exceed the hematite performances, with a ∼ 1 mA· ${\mathrm{cm}}^{-2}$ current density in an extremely thin 6 nm film. These results contradict recent claims of γ‐${\mathrm{Fe}}_{2}$ $O_{3}$ unsuitability [22]. The apparent inactivity on samples prepared via hydrothermal syntheses likely stems from uncontrolled magnetite formation and surface reconstruction, creating excessive recombination sites, whereas our Ni‐doped epitaxial films eliminate such defects through atomic‐scale ordering. This discrepancy highlights the importance of synthesis techniques that offer superior control on samples stoichiometry, crystallinity and morphology, accompanied by adequate analysis capable to assess phase purity and even minor changes in the active sites local structure, such as XAS. Using *operando* XAS, we confirmed how ${\mathrm{Fe}}_{Oh}$ are the active sites for the OER. In particular, using a novel X‐ray absorption pump‐and‐probe method, we were able to probe the light‐driven surface species kinetics. Our results suggest short‐lived highly valent Fe species formed upon oxidation of the hydroxyl absorbed to ${\mathrm{Fe}}_{Oh}$ sites, followed by O–O bond formation in the form of metastable superoxo surface species, which accumulates at the surface in a ∼ 300 ms timescale during solar illumination. Further investigations will be required to confirm our results, but we hope that our work will stimulate the design of new synthesis methods to obtain highly pure single‐phase nanostructured doped maghemite. Such high surface‐to‐bulk systems also facilitate systematic *operando* XAFS characterizations by enhancing signal‐to‐noise ratio, paving the way to more active OER photocatalysts.

## Author Contributions

F.P. performed all measurements, calculations, data curation and writing; H.M. sample preparation, measurements, funding; E.F. conceptualization, setup development, measurements, methodology, writing; A.B. XAS measurements and conceptualization. All authors contributed to the final editing of the manuscript.

## Conflicts of Interest

The authors declare no conflicts of interest.

## Supporting information


**Supporting File**: advs74828‐sup‐0001‐SuppMat.pdf.

## Data Availability

The data that support the findings of this study are available from the corresponding author upon reasonable request.
