# Supplementary material for: Tuning Defects in Ni‐Doped Maghemite for Enhanced Solar Driven Oxygen Evolution: Insights From Operando X‐Ray Spectroscopies
Source: Adv Sci (Weinh). 2026 Mar 31;13(30):e24386. doi: 10.1002/advs.202524386 (PMC13248805; doi:10.1002/advs.202524386)
Supplement: Supplementary file 1 — Supporting File: advs74828‐sup‐0001‐SuppMat.pdf. [file ADVS-13-e24386-s001.pdf]

Tuning Defects in Ni-Doped Maghemite for Enhanced Solar Driven Oxygen Evolution: Insights from *Operando* X-ray Spectroscopies.
# - Supporting Information –

Francesco Paparoni,[1,2] Hélène Magnan,[3] Antoine Barbier,[3] Andrea Zitolo,[2] Andrea Di Cicco,[1] Seyed Javad Rezvani,[1] and Emiliano Fonda[2]

1)Sezione di Fisica, School of Science and Technology, University of Camerino, Camerino, Italy.

2)Synchrotron SOLEIL, Départementale 128, 91190 Saint-Aubin, France.

3) Université Paris-Saclay, CEA, CNRS, Service de Physique de l'Etat Condensé, F-91191 Gif-sur-Yvette, France

S1. Reference XAS spectra

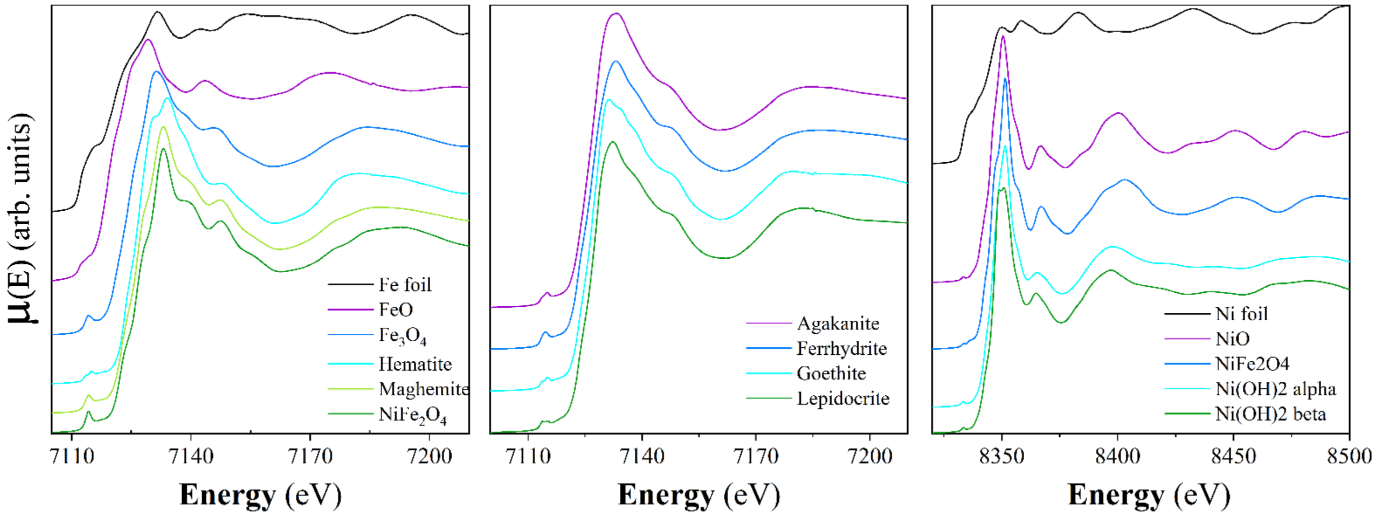

Figure S1. Reference Fe oxide (left), Fe hydroxide (center), and Ni oxide and hydroxide (right) XAS spectra collected for this project.

During this project, several useful reference spectra were collected at the SAMBA beamline to compare with our sample. All references were prepared as pellets with a 10 mm diameter, mixing 10 mg of high-purity powder sample (Sigma Aldrich, purity >99.6%) from commercial powder with 40 mg of graphite. All XAS measurements were carried out at the SAMBA beamline in transmission mode. Several Fe and Ni oxides, with distinct oxidation states or structures, were probed at the Fe and Ni K-edges to be compared with the Ni-doped maghemite sample spectra to understand the spectral evolution to be expected in the case of electronic or structural changes and confirm the sample stability. Several Fe and Ni hydroxides were also measured to understand the spectroscopic effect of the hydroxyl absorption. All the resulting Fe and Ni K-edges XAS spectra are shown in Figure S1.

S2. Mylar window.

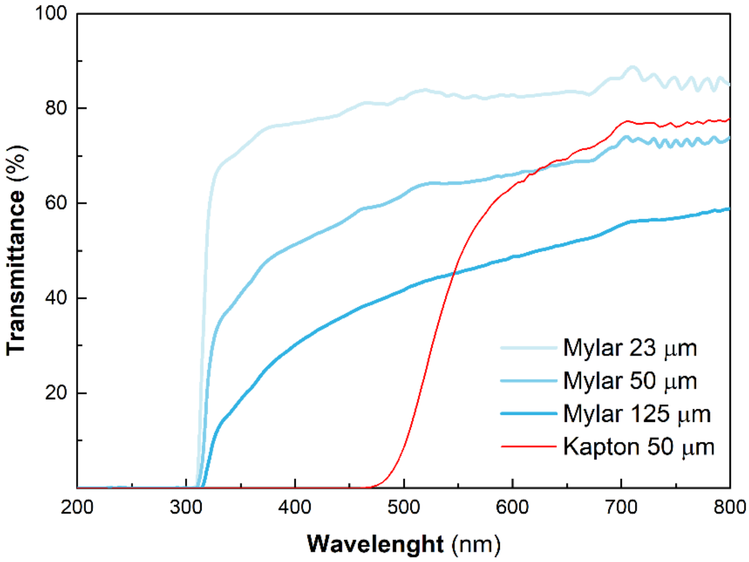

Figure S2. UV-VIS measurement on several Kapton and Mylar widows with different thickness.

As shown in figure 2S, the Mylar window offer much lower near-UV absorption compared to Kapton, in the region were samples shown the highest solar-to-hydrogen conversion efficiency. A 50 micron mylar window was hence used, since the 23 micron one was found to be too fragile to hold for several hours. The 50 micron mylar window was found to hold for around 12-18 hours of continuous use under the beam, which arrive to even a full day with a non-completely focused beam (1 mm diameter) and by slightly moving the beam position after few hours.

S3. Morphological Analysis

The crystallographic structure and the morphology of the as-prepared Ni-doped maghemite films were studied, respectively, from RHEED (3S) and AFM (4S). RHEED analysis confirms the formation of a crystalline spinel structure with 111 orientation, with no apparent modification induced by the different Ni concentrations.

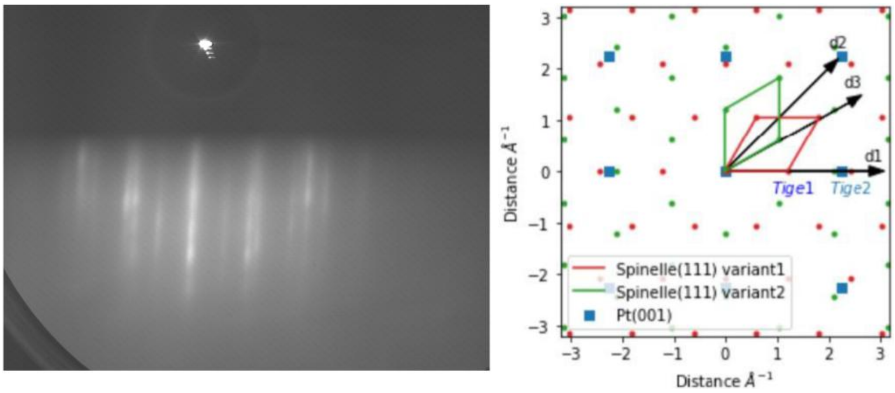

Figure S3. (left) RHEED patterns along a particular azimuth corresponding to one of the reciprocal space surface diffraction directions of lowest Miller indexes. (right) corresponding surface reciprocal lattices for γ-Fe_2O_3 (111) /Pt (111). The elementary cell in the reciprocal space is in the same color as the corresponding reciprocal lattice.

Regarding surface roughness, AFM images confirm a smooth, homogeneous surface for all samples, with no clear modulations caused by the Ni doping concentration. We estimated sample's roughness between 2 and 3 nm, resulting in roughness factors extremely close to 1, as in perfectly smooth surfaces.

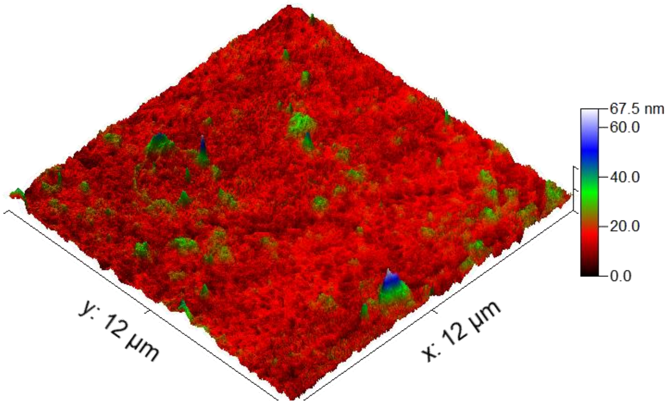

Figure S4. . AFM 3D topography image of a sample with 25% Ni content

S4. Effect of Thickness on the photocurrent.

As suggested by Lopes et al. [1], for catalyst thicknesses below the space charge layer width, the semiconductor thickness limits the photo-response, and thus, the photocurrent generated is lower. Comparing his results with other works on Fe2O3 photoanodes[2], we can expect a space charge layer in the 20-30 nm range. Our results show that our sample's photocurrent increases with the film thickness, confirming that our films have thicknesses below the space charge layer width. While pristine maghemite appears less performant than the reference Ti-doped hematite thin films, our results show that the sample with optimal Ni doping outperforms the doped hematite sample.

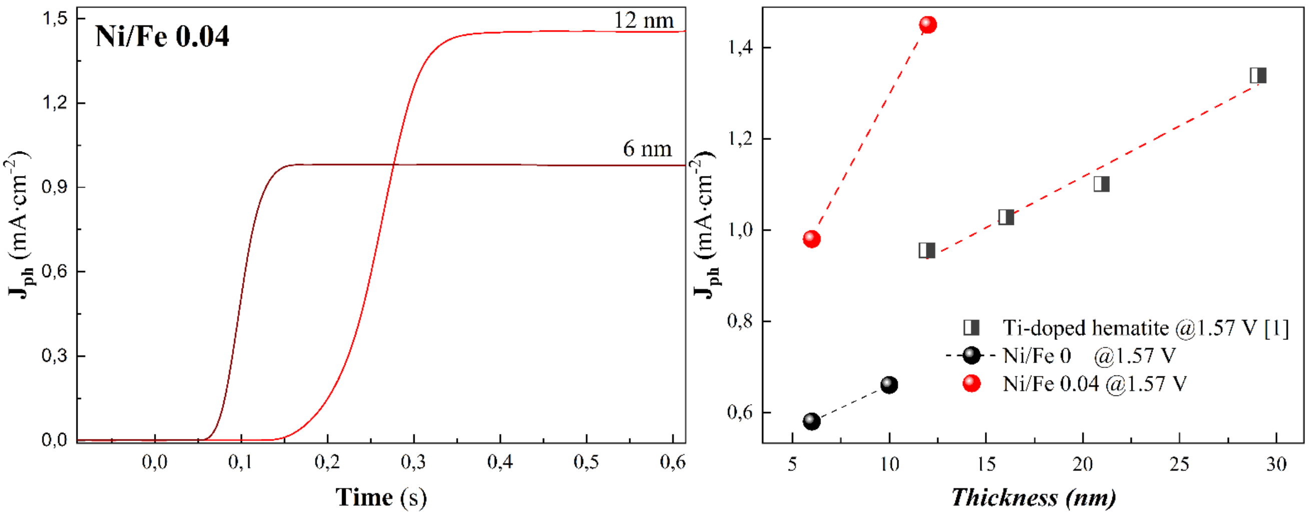

Figure S5. Photocurrent measured at 1.57 V vs RHE under 4.5 sun illumination on Ni-doped maghemite films with identical doping and different film thicknesses (left). On the right, samples photocurrent vs film thickness compared with the results of ref 2 for 2% Ti-doped Hematite in similar experimental conditions.

[1] Lopes et al., «Hematite Photoelectrodes for Water Splitting».
[2] Rioult et al., «Single Crystalline Hematite Films for Solar Water Splitting».

Table S1. Literature comparison.

Photoanode [ref] Our samples	Photocurrent [mAcm^{-2}]	Applied Potential [V vs RHE]	Thickness [nm]	Illumination Power [Sun]	Electrolyte
4.6% Ni doped **Maghemite** (111)	1.0	1.57	6	4.5	0.1 M KOH
4.6% Ni doped **Maghemite** (111)	1.45	1.57	12	4.5	0.1 M KOH
4.6% Ni doped **Hematite** (111)	0.21	1.57	6	4.5	0.1 M KOH
5% Ti-doped **Hematite** (001) [1]	8	1.57	40	1	0.1 M NaOH
2% Ti-doped **Hematite** (001) [2]	1.3	1.57	30	1	0.1 M NaOH
W-doped **Bi_2MoO_6** Nanoflakes [3]	1.81	1.23	?	1.1	0.1 M Na_2SO_4
**$NiFeO_x/Ta_3N_5/SiO_2$** [4]	5.9	1.23	?	1	0.2 M KH_2PO_4
**W-doped**					

1. H. Magnan, Appl. Phys. Lett. 101, 133908 (2012), https://doi.org/10.1063/1.4755763
2. M. Rioult, J. Phys. Chem. C (2014) 118,3007-3014
3. M. Chakraborty, Sustainable Energy and fuels, 3, 2020 https://doi.org/10.1039/C9SE00796B
4. T. Higashi, Phys. Chem. Chem. Phys., 2023, 25, 20737 https://doi.org/10.1039/d3cp02563b

S5. XRF ANALYSIS

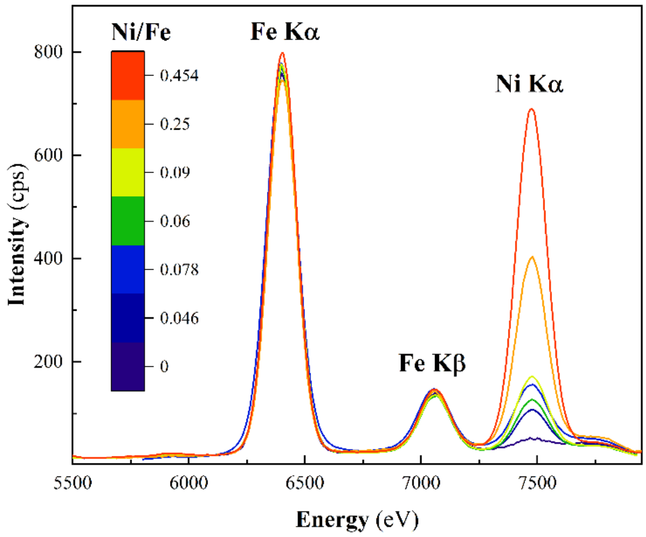

	Ni/Fe (%)	err %
pt36e	0	0.0005
pt44d	2	nd
pt43e	4.6	0.1
pt35e	6.1	0.1
pt40d	7.8	0.1
pt42g	9.0	0.2
pt37b	25.8	0.3
pt34f	45.4	0.4

Figure S6. XRF spectra collected at the SAMBA beamline with identical acquisition conditions with 10 keV incoming energy.

S6. EXAFS ANALYSIS

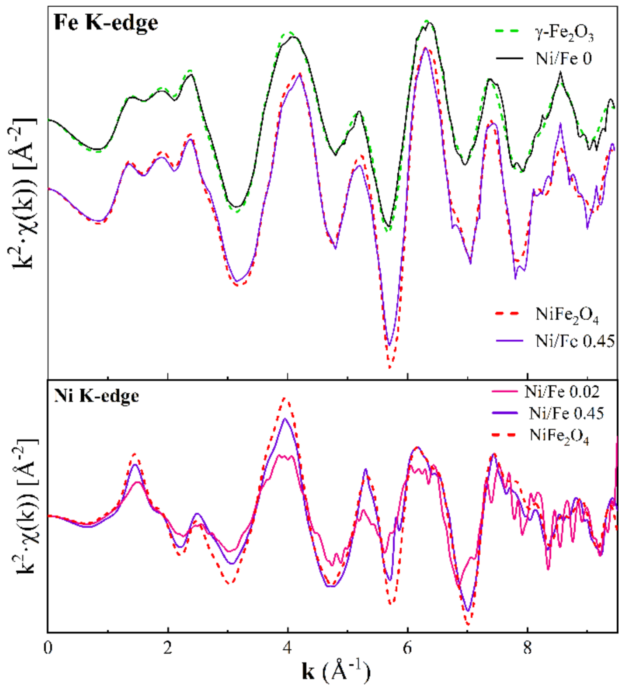

Figure S7. Comparison between the Fe K-edge (top) and Ni K-edge (bottom) EXAFS spectra of selected samples with maghemite (dashed green) and NiFe2O4 (dashed red) references.

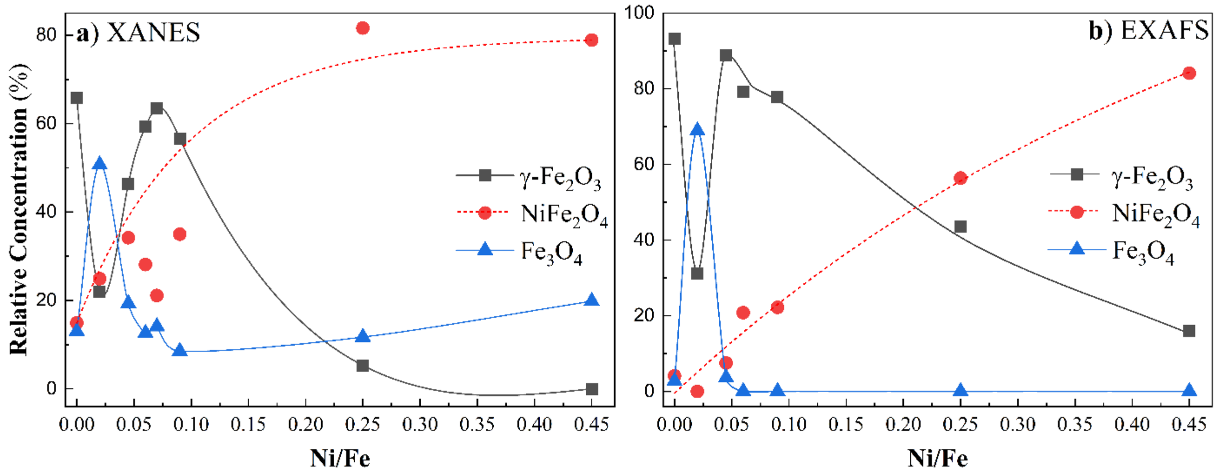

Figure S8. Linear combination fitting of the Fe K-edge XANES (a) and k-space EXAFS (b) spectra as a function of the Ni concentration.

Table S2. Best-fit results of the Fe (top) and Ni (bottom) EXAFS K-edges. Fe$_{Td}$ and Fe$_{Oh}$ indicate the photoabsorbers in tetrahedral and octahedral sites, respectively. Debye-Waller factors (σ^2) have been multiplied by a 10^2 factor to improve visualization.

Fe K-edge	Ni/Fe 0.04			Ni/Fe 0.25		
**Bond**	**CN**	**R(Å)**	**σ^2(Å) ·10^2**	**CN**	**R(Å)**	**σ^2(Å) ·10^2**
**Fe$_{Oh}$-O**	6	2.02 ± 0.03	0.9 ± 0.3	6	2.00 ± 0.02	0.22 ± 0.08
**Fe$_{Td}$-O**	4	1.91 ±0.03	0.3± 0.1	4	1.85 ± 0.02	0.13 ± 0.05
**Fe$_{Oh}$-Fe$_{Oh}$**	5	3.05 ± 0.02	0.21± 0.08	5	3.03 ± 0.03	0.27 ± 0.08
**Fe$_{Oh}$-Ni**	1 ± 0.1	3.02 ± 0.03	0.6 ± 0.1	1.4 ± 0.2	2.97 ± 0.03	0.9 ± 0.2
**Fe$_{Td}$-Ni**	3 ± 0.3	3.54 ± 0.04	0.4 ± 0.1	4.2 ± 0.6	3.57 ± 0.05	0.3 ± 0.1
**Fe$_{Td}$-Fe$_{Oh}$**	10	3.51 ± 0.05	1.9 ± 0.3	9	3.47 ± 0.03	1.1 ± 0.1
**Fe$_{Oh}$-Fe$_{T}$d**	6	3.51 ± 0.05	1.9 ± 0.3	6	3.47 ± 0.03	1.1 ± 0.1
**Fe$_{Td}$-Fe$_{Td}$**	4	3.79 ± 0.09	0.6 ± 0.1	4	3.77 ± 0.04	0.2 ± 0.2
***Fe$_{Td}$-Fe$_{Oh}$**	3	4.8 ± 0.1	1.3 ± 0.2	3	5.2 ± 0.2	0.90 ± 0.1
Ni K-edge						
**Shell**	**CN**	**R(Å)**	**σ^2(Å) ·10^2**	**CN**	**R(Å)**	**σ^2(Å) ·10^2**
**Ni-O**	6	2.10 ± 0.01	1.1 ± 0.1	6	2.05 ± 0.02	1.1 ± 0.1
**Ni - Fe$_{Oh}$**	3	3.04 ± 0.03	0.20 ± 0.04	3	2.96 ± 0.02	0.08 ± 0.04
**Ni - Fe$_{Td}$**	3	3.50 ± 0.03	0.60 ± 0.10	3	3.50 ± 0.03	0.20 ± 0.05

S7. XPS

XPS Fe 2p fitting routine was carried out fixing all components position according to the work of Y. He et al., Journal of Alloys and Compounds 917 (2022) 165494, with values indicated in the manuscript. The FWHM component of the $Fe^{3+}_{Oh}(2P_{3/2})$, here called FWHM_0, was fitted in a narrow range (2.29-2.39 eV) around the value used in the same work. All other FWHM components were fixed to the FWHM_0 fitted value and multiplied for an experimental factor, which was estimated again from the results of Y. He et al., and optimized for our data. As a result, we used FWHMs of 2.9 ± 0.2 eV for $Fe^{3+}_{Td}(2P_{3/2})$, 1.65 ± 0.05 eV for $Fe^{2+}(2P_{3/2})$, 3.3 ± 0.1 eV for Fe^{2+} satellite, 4.8 ± 0.2 eV for the Fe^{3+} satellite. For the 2p ½ components, we obtained 2.1 ± 0.1, 3.2 ± 0.2 and 3.4 ± 0.2 eV for Fe^{2+}, Fe^{3+}_{Oh} and Fe^{3+}_{Td} respectively. Restraints were also inserted in the areas parameters. In agreement with the Fe 2p level degeneracy, the areas of the 2p ½ components were fixed to half of the 2p 3/2 ones. To avoid Fe^{3+}_{Td} underestimation, the Fe^{2+} satellite intensity was also restrained to stay below 0.7 times the area of the $Fe^{2+}(2P_{3/2})$ component. Initial refinement was carried out on the pristine sample, keeping the octahedral to tetrahedral ratio at the stoichiometric value, then the ratio of the two components was freed on the other samples. Asymmetric Lorentzian with gaussian broadening (LF 1,1,25,280) line shapes were used in all cases. Fitted curves are shown in Figure S9, with relative fitting results.

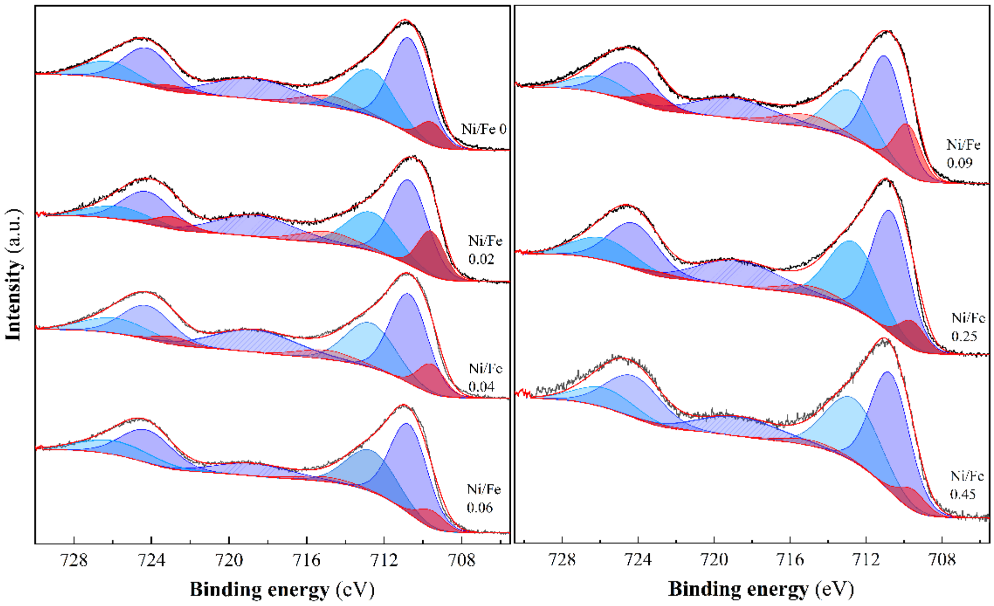

Ni/Fe	FeOh/FeTd	Fe2+(%)
0	1.66 ± 0.09	9.8 ± 1.3
0.02	1.67 ± 0.08	23.2 ± 1.5
0.04	1.54 ± 0.06	14 ± 1
0.06	1.65 ± 0.09	8.6 ± 1.1
0.09	1.57 ± 0.05	10 ± 1
0.25	1.36 ± 0.04	9.2 ± 0.09
0.45	1.34 ± 0.07	7.1 ± 1.1

Figure S9. Fe 2p XPS fitted spectra for samples with distinct Ni/Fe ratios. On the right, fitting results table and components.

To better understand the surface hydroxylation, XPS analysis at the O 1s region were conducted on two selected sample with low (0.02 Ni/Fe) and high (0.2 Ni/Fe) Ni content. Samples were measured after the *operando* experiments at the SAMBA beamline, where they were illuminated by chopped light while at 1.57 V vs RHE. Finally, samples were cleaned by DI water rinsing and subsequent annealing in air at 350 °C and measured again to confirm the removal of surface adsorbed water and OH^-. Finally, samples where immersed in a 0.1 M NaOH solution (pH 13) for 40 min before being dried with compressed air and immediately inserted again in the XPS chamber. Our results are shown in Fig. S10.

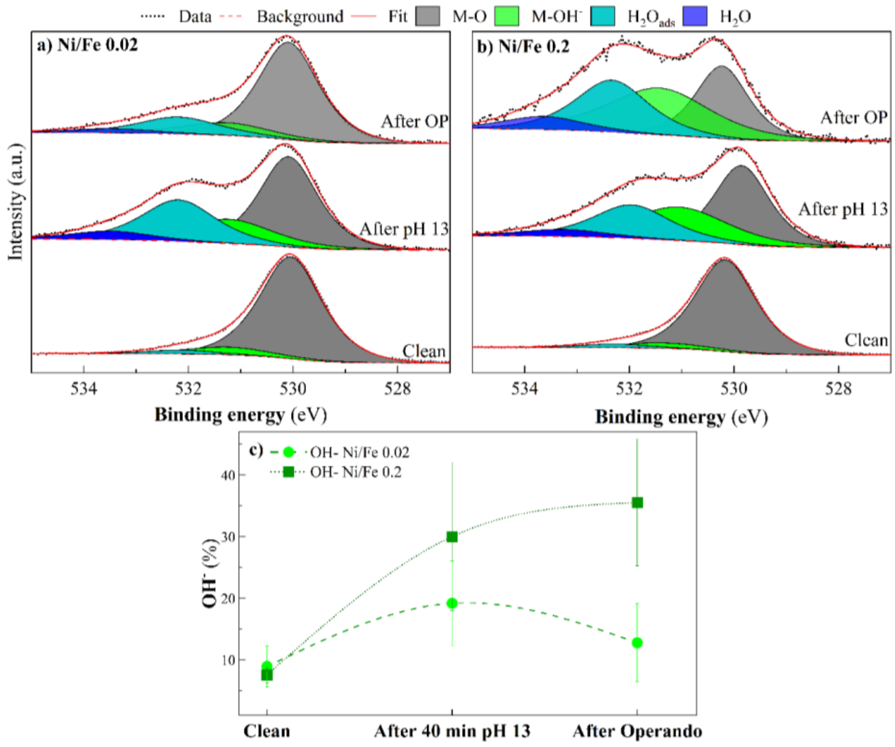

Figure S10. Fitted O 1s XPS spectra collected from samples with 0.02 (a) and 0.2 (b) Ni/Fe after cleaning (clean), electrolyte immersion (after pH 13) and after the operando experiment at SAMBA. Below (c) the surface adsorbed OH^- estimated from the fit at the different stages.

S8. FEXRAP and XAS under Illumination

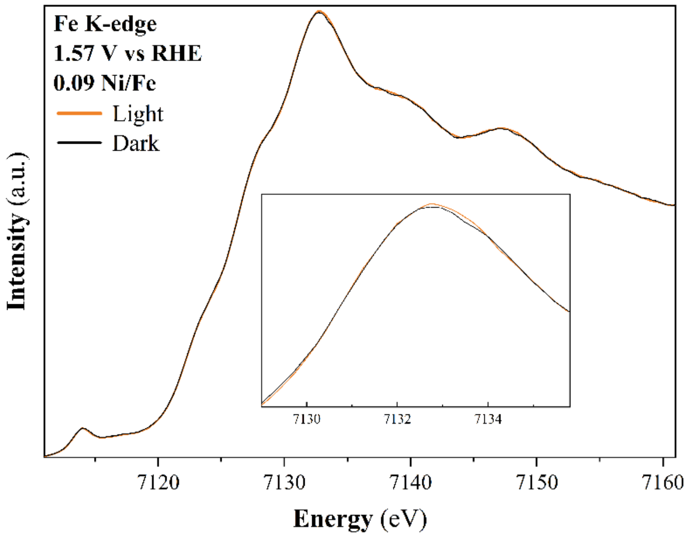

Figure S11. XANES data collected under PEC operando conditions, while applying a 1.57 V potential in CA mode, under dark (black) and 4.5 sun illumination (orange). A magnified views of the white line changes is shown in the inset.

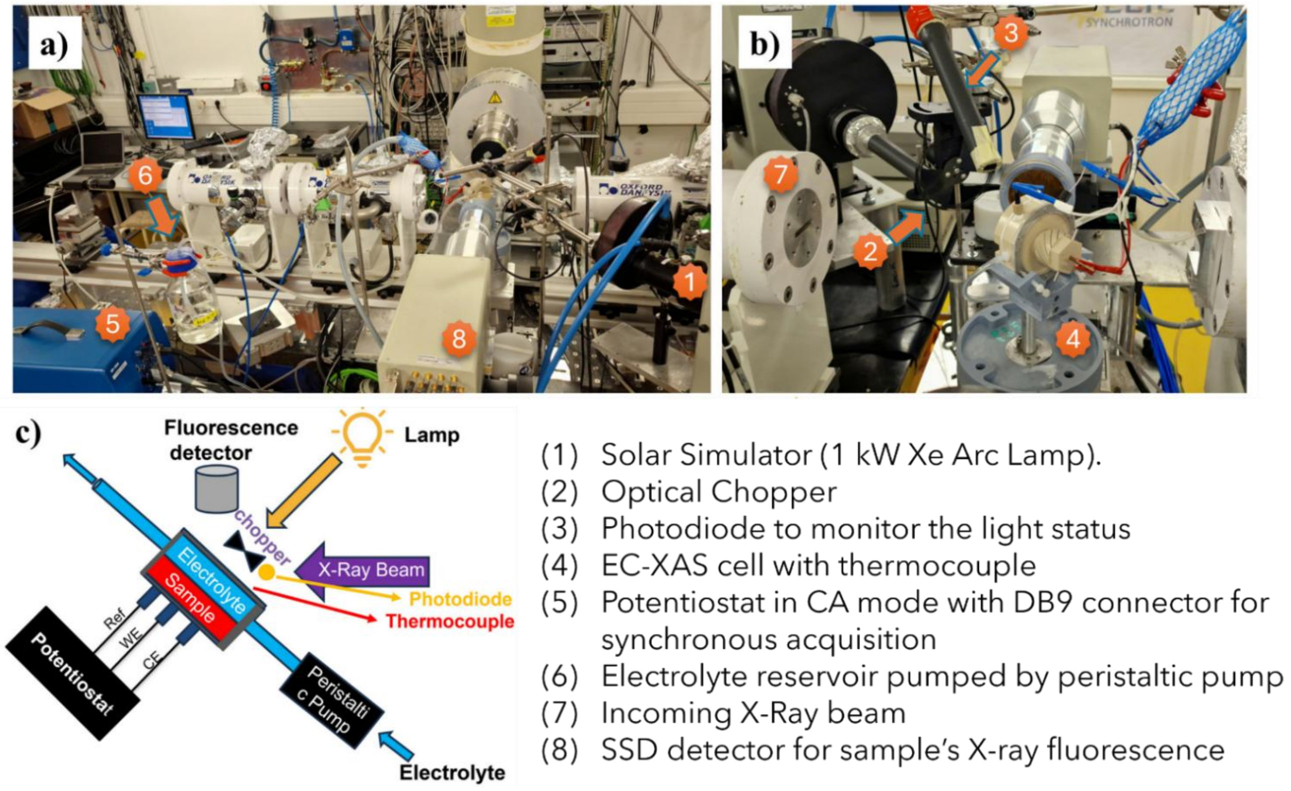

Figure S12. Photo of the FEXRAP setup on the SAMBA beamline (a) with magnified view on the operando cell (b). A scheme is given in fig. c.

Sample temperature read from the thermocouple was folded as for the FEXRAP signal. The highest temperature increase after 0.5 s of light on is less than 0.3 °C, as shown in Fig. S13, independently from the applied potential and the presence of FEXRAP signal. Figure also shows that the same signal is not observed at other energies. X-ray fluorescence data *F(t)* were normalized as % variation of the absorption coefficient Δμ(%) with respect to the average fluorescence intensity without illumination B_d:

$$\Delta\mu(\%) = 100 * \frac{F(t) - B_d}{B_d}$$

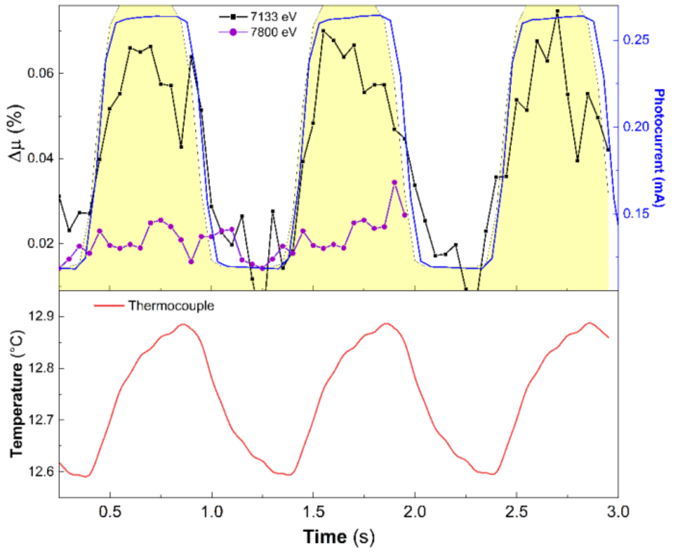

Figure S13. FEXRAP signal collected from the optimal 0.04 Ni/Fe sample at 0.6 V Ag/AgCl at 7133 eV (black squares) and 7800 eV (purple dots) Vs photocurrent (blue) and sample temperature (red, bottom).

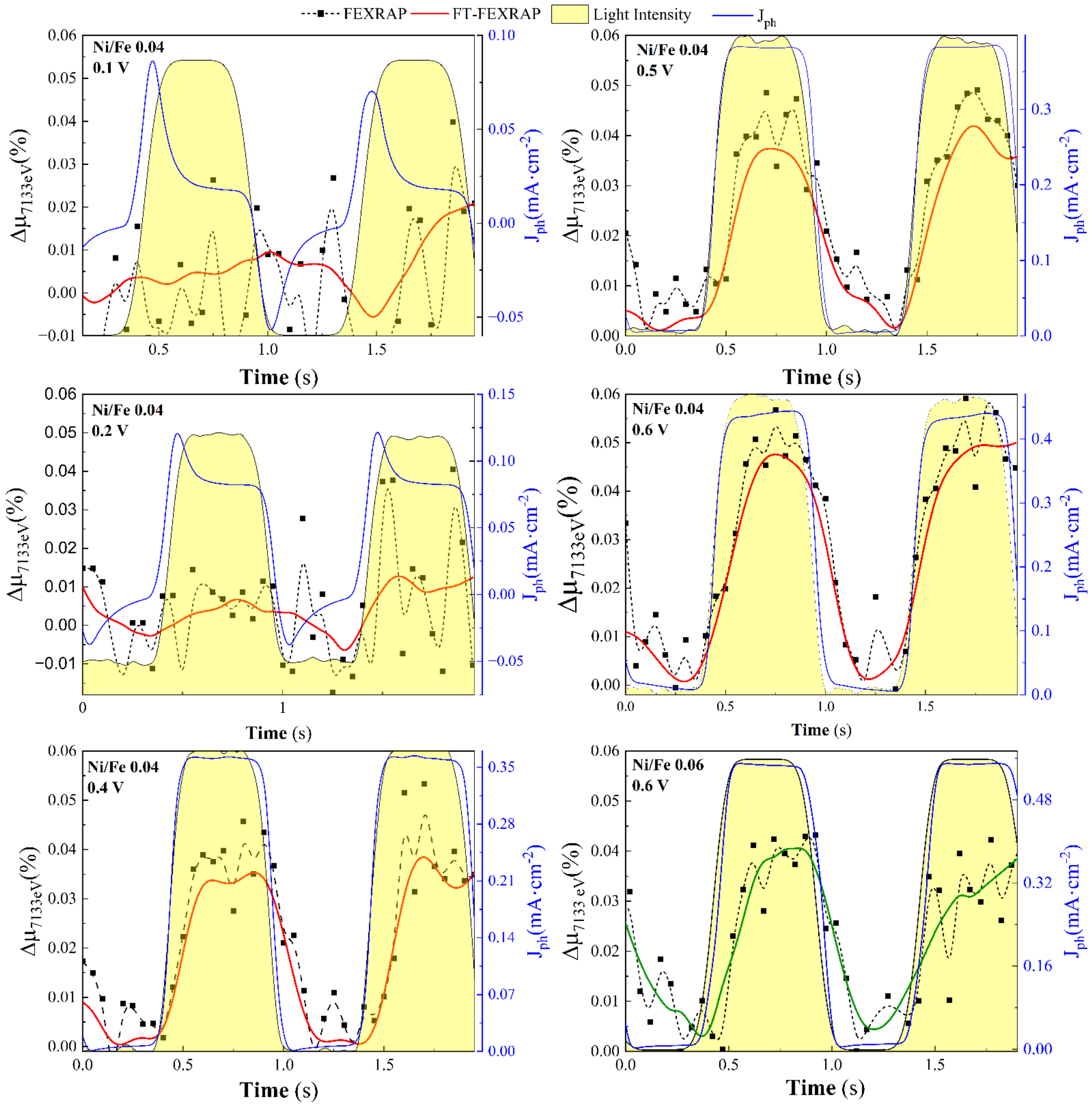

Figure S14. FEXRAP measurements at the Fe K-edge white line on the optimal 0.04 Ni/Fe sample under identical 2 sun, 1 Hz chopped illumination, while at different applied powers. The % difference in absorption coefficient is in black dashed line, with relative 2-point Fourier transformed smoothed curve in red. The blue curve shows the simultaneously collected photocurrent density, while in yellow the illumination status. At the bottom right, the graph relative to another sample with 0.06 Ni/Fe ratio, with highlighted FT-FEXRAP data in green.

The time constant τ of the signal raising $\Delta\mu_r(t)$ and decay $\Delta\mu_d(t)$ were obtained from the typical exponential relations shown below, with two constant (μ_0 offset kept to 0 and t_1 being the time at which the light was switched off) and two fitted variables: pre-exponential factor (A) and time constant.

$$\Delta\mu_r(t) = \mu_0 + A * \left[1 - e^{-\frac{t}{\tau}}\right]$$

$$\Delta\mu_d(t) = \mu_0 + A * \left[e^{-\frac{t-t1}{\tau}}\right]$$

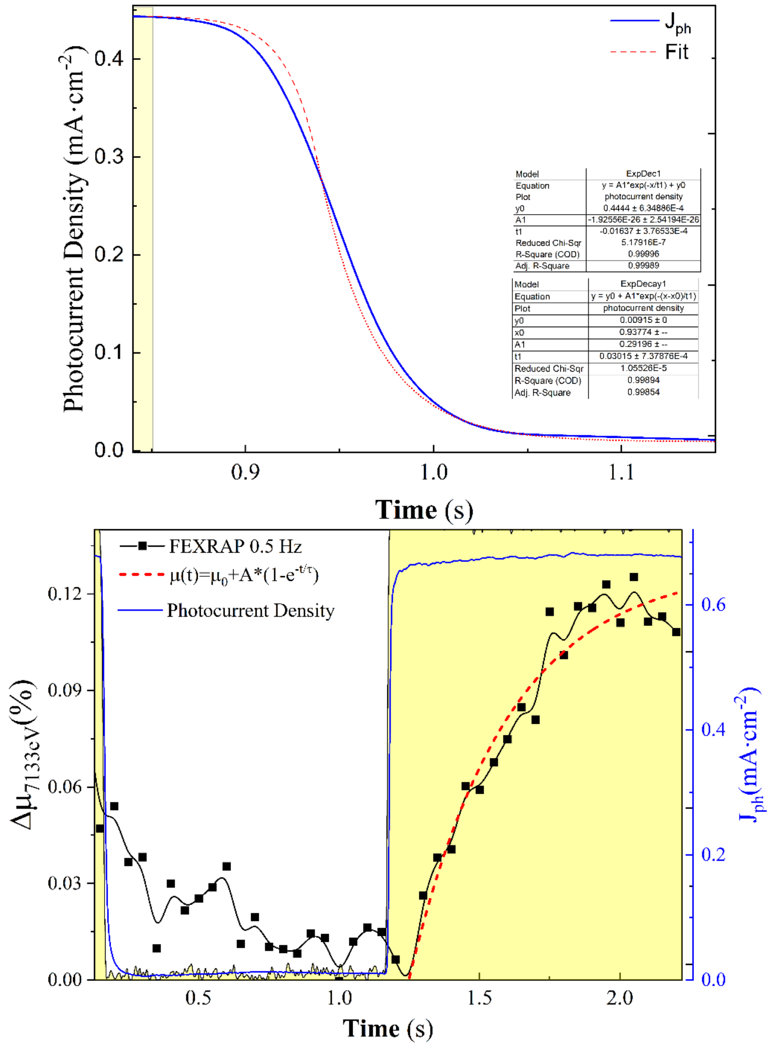

Figure S15. Photocurrent decay vs time. In yellow is highlighted the light on state. Below, Single-period of the FEXRAP signal collected from the optimal 6 nm thick 0.04 Ni/Fe sample with 0.5 Hz chopper period. In red, the exponential fit to estimate the raising time.

S9. FEFF simulations

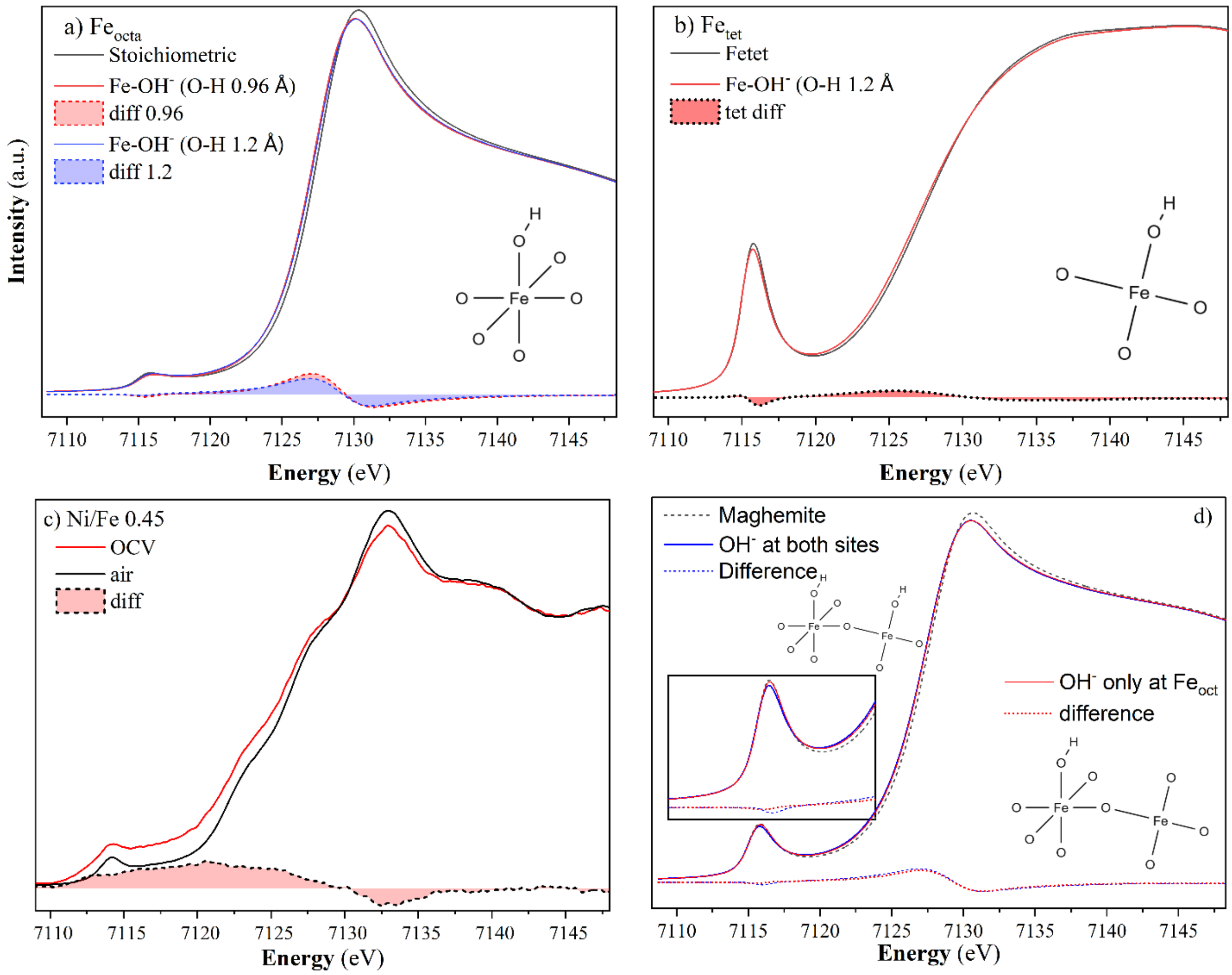

Figure S16. Calculated Fe K-edge spectra relative to the Fe_{oct} (a) and Fe_{tet} (b) photoabsorbers using a 2.5 angstrom cluster only including the first O coordination shell surrounding the photoabsorber. Spectral differences compared to the stoichiometric case (black curve) for distinct O-H bond length typically associated with hydroxyl are shown. The molecular configurations are sketched in the insets. C) Experimental modulations at the Fe K-edge in the samples with highest Ni content. D) Sum of octahedral and tetrahedral contributions in the case of double site absorption and only octahedral site absorption.

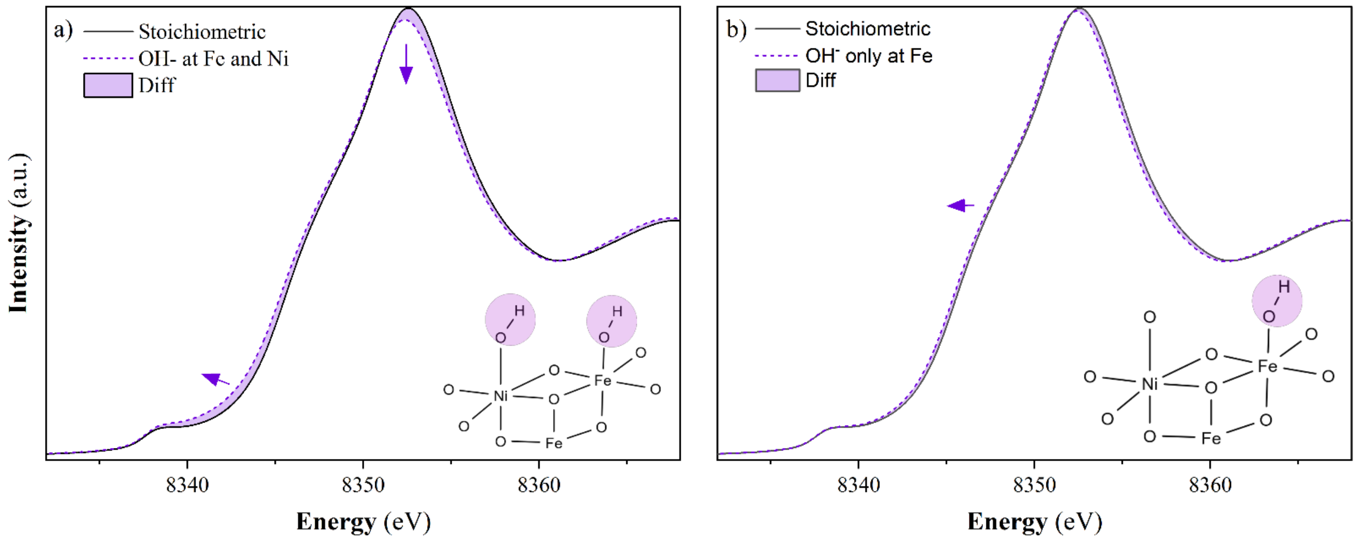

Figure S17. Calculated Ni K-edge spectra using a 4 angstrom cluster around a Ni photoabsorber in a Fe octahedral site of maghemite, before and after OH^- absorption at both Ni and Fe octahedral sites (a) and just at the Fe octahedral sites (b).

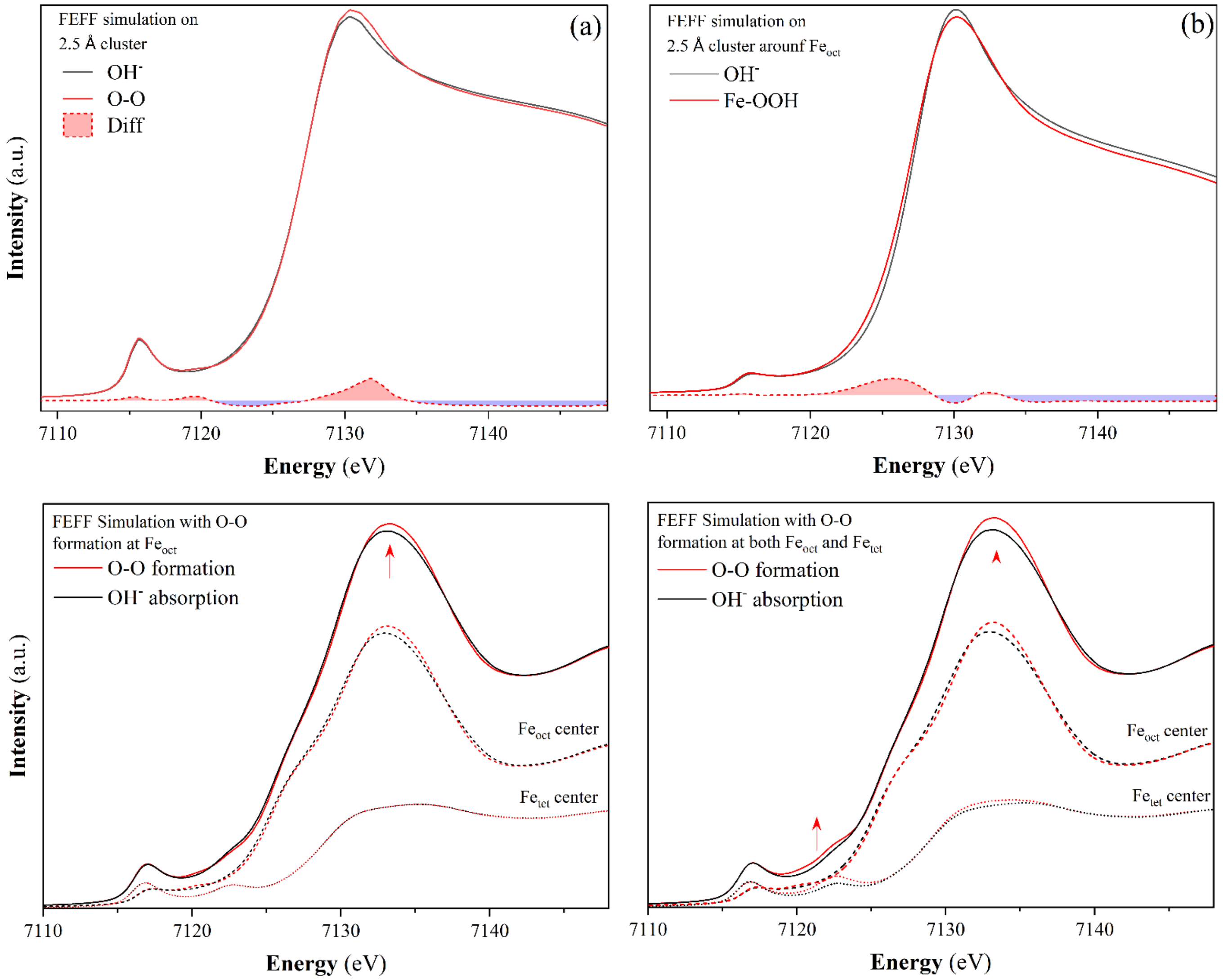

Figure S18. (a-b) Calculated Fe K-edge spectra using a 2.5 angstrom cluster. In black, the spectra calculated in the case of OH^- absorption at a first shell O site and to second shell O site. In red, (a) the spectra obtained removing hydroxyl bond at the octahedral sites and replacing them with an O-O bond or an O-O-H bond (b). Below, Fe K-edge spectra using a 4 angstrom cluster around a Fe at tetrahedral site (dotted lines) and Fe octahedral site (dashed lines), weighted averaged. In red, O-O bond formation at just the first O shell of the Fe octahedral site (left) or also at the tetrahedral site (right).

Operando XAS.

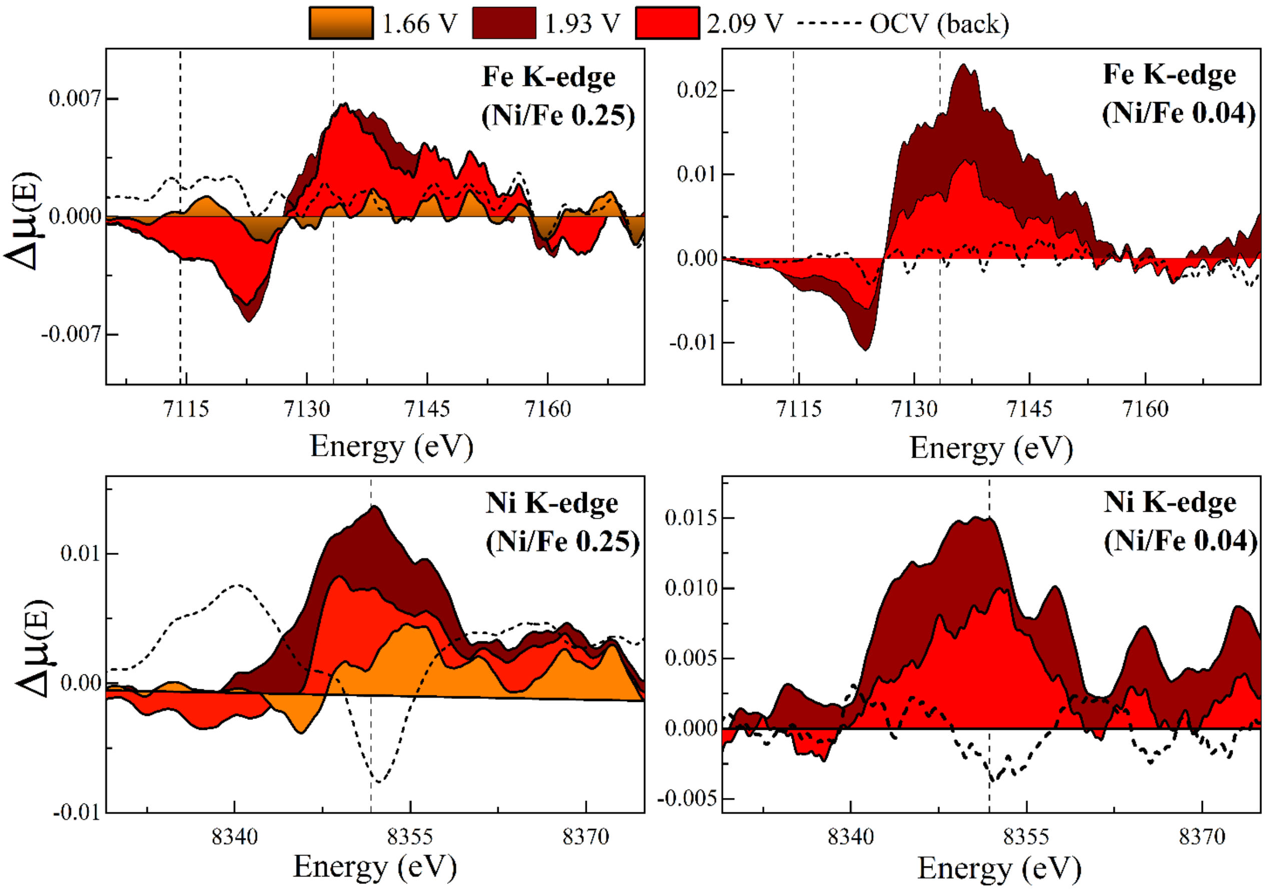

Figure S19. Spectral differences between the operando XAS spectra acquired at each potential point and the one acquired at OCV are plotted below the XANES as colored areas. ''OCV (back)'', taken at open circuit potential after the measure at 2.09 V, is indicated by dashed curves. Vertical dash lines highlight the pre-edge and white line positions.
